# HIV induces airway basal progenitor cells to adopt an inflammatory phenotype

**DOI:** 10.1038/s41598-021-82143-1

**Published:** 2021-02-17

**Authors:** Nancy P. Y. Chung, K. M. Faisal Khan, Robert J. Kaner, Sarah L. O’Beirne, Ronald G. Crystal

**Affiliations:** 1 Department of Genetic Medicine, Weill Cornell Medical College, 1300 York Avenue, Box 164, New York, NY 10065 USA; 2 Department of Medicine, Weill Cornell Medical College, New York, NY USA

**Keywords:** Cell biology, Diseases

## Abstract

Despite the introduction of anti-retroviral therapy, chronic HIV infection is associated with an increased incidence of other comorbidities such as COPD. Based on the knowledge that binding of HIV to human airway basal stem/progenitor cells (BC) induces a destructive phenotype by increased MMP-9 expression through MAPK signaling pathways, we hypothesized that HIV induces the BC to express inflammatory mediators that contribute to the pathogenesis of emphysema. Our data demonstrate that airway BC isolated from HAART-treated HIV^+^ nonsmokers spontaneously release inflammatory mediators IL-8, IL-1β, ICAM-1 and GM-CSF. Similarly, exposure of normal BC to HIV in vitro up-regulates expression of the same inflammatory mediators. These HIV-BC derived mediators induce migration of alveolar macrophages (AM) and neutrophils and stimulate AM proliferation. This HIV-induced inflammatory phenotype likely contributes to lung inflammation in HIV^+^ individuals and provides explanation for the increased incidence of COPD in HIV^+^ individuals.

## Introduction

With the development of highly active anti-retroviral therapy (HAART), the survival rate of HIV-infected individuals and life span has been dramatically improved^1,2^. However, this has been accompanied by an increased risk for other co-morbidities, including the development of chronic obstructive pulmonary disease (COPD), manifesting as emphysema^3–8^. Even with effective anti-retroviral therapy that normalizes blood viral load and CD4^+^ cell levels, COPD is more prevalent in the HIV-infected population^3,5–12^. Several factors have been implicated in the increased susceptibility to COPD in this population including higher prevalence of smoking among HIV^+^ individuals, exaggerated lung inflammation (e.g., mediators released by alveolar macrophages and CD8^+^ T cells), adverse effects of long-term anti-retroviral therapy and the direct effect of HIV-related proteins such as Tat and Env (gp120) on lung cells^13–19^.

Although the mechanisms of the development of COPD in association with HIV infection are not clear, it is recognized that in HIV-infected individuals: (1) the lung is an HIV reservoir^20–29^; (2) HIV infection is associated with chronic lung inflammation dominated by increased numbers of activated alveolar macrophages and CD8^+^ T cells which release various inflammatory mediators in the local milieu^15,20,30–35^; and (3) chronic inflammation in the lung mediates lung injury and disordered repair typical of COPD^11,36–38^.

While lung inflammation likely plays a central role in the pathogenesis of HIV-related development of COPD, the source of the signals initiating the enhanced inflammation has been focused on alveolar macrophages, the lung representative of the mononuclear phagocyte system^20,39–42^. Based on our recent observation that airway basal cells (BC) have receptors for HIV and HIV binding to BC induces the BC to express and release matrix metalloproteinase 9 by activating the MAPK pathway, a pathway closely linked to NFκB and transcription factors for expression of inflammatory mediators^43–46^, we hypothesized that the consequences of HIV binding to BC may be more extensive, and that HIV may induce BC to adopt an “inflammatory phenotype,” with expression and release inflammatory cytokines, contributing to the enhanced inflammatory milieu that characterizes the HIV^+^ lung. To assess this hypothesis in vivo, small airway BC isolated from HAART-treated HIV^+^ nonsmokers and normal healthy HIV^-^ nonsmokers were used to compare the secretion of inflammatory mediators. To validate these ex vivo observations in vitro, we examined gene expression, secretion and function of cytokines and mediators released from airway BC after in vitro exposure to HIV using TaqMan PCR, ELISA, Western analysis and migration and cell proliferation assays.

## Methods

### Assessment of airway BC from HAART-treated HIV^+^ and healthy HIV‾ nonsmokers

All individuals enrolled in this study were evaluated at the Department of Genetic Medicine Clinical Research Facility, using Weill Cornell Medicine Institutional Review Board-approved clinical protocols. All experiments were performed in accordance with relevant guidelines and regulations. Informed consent was obtained from each individual prior to enrollment of this study. Individuals underwent an initial screening evaluation including history, complete physical exam, blood studies, urine analysis, chest X-ray, pulmonary function tests, and electrocardiogram. Individuals with any significant prior use of addictive drugs in the previous 6 months were excluded and required to meet all criteria established for the study (Supplemental Experimental Procedures). Blood studies included a complete blood count, coagulation parameters, serum electrolytes, liver and kidney function tests, serum evaluation for human immunodeficiency virus antibodies, HIV-1 viral load, CD4 count, hepatitis profile (A, B, and C), anti-nuclear antibodies, sedimentation rate, and rheumatoid factor^47^. Pulmonary function tests were carried out according to American Thoracic Society guidelines^48–51^. All HIV‾ healthy nonsmokers (n = 4) and HAART-treated HIV^+^ nonsmokers (n = 4) had a normal screening evaluation, normal pulmonary function tests and chest X-ray, and a negative urine screen for smoking. Information of HAART regimen taken by HIV^+^ nonsmokers was also provided (see Table 1 for details). Special handling precautions were taken by lab personnel when working with cells isolated from HIV^+^ individuals.

**Table 1:** Demographics of normal and HAART-treated HIV^+^ Nonsmokers.

Sample ID	Age	Gender/ethnicity	HIV status	Phenotype	Viral load (copies/ml)	Blood CD4 (cell # per µl)	HAART regimen
1	22	M (African)	−	NS	NA	NA	NA
2	50	M (African)	−	NS	NA	NA	NA
3	43	F (African)	−	NS	NA	NA	NA
4	42	F (Caucasian)	−	NS	NA	NA	NA
5	53	F (other)	+	NS	Detected, < 20	1106	Elvitegravir/Cobicistat/Emtricitabine/Tenofovir alafenamide (Genvoya)
6	19	M (African)	+	NS	Detected, < 20	500	Efavirenz, emtricitabine, and tenofovir disoproxil fumarate (Atripla)
7	43	M (Hispanic)	+	NS	Not detected	1080	Elvitegravir/Cobicistat/Emtricitabine/Tenofovir alafenamide (Genvoya)
8	45	M (African)	+	NS	Not detected	750	Emtricitabine, rilpivirine, and tenofovir disoproxil fumarate (Complera)

*NS* nonsmoker, *NA* not applicable.

Small airway epithelial cells were collected by fiberoptic bronchoscopy by brushing as previously described^52–54^. After routine anesthesia, a 2 mm disposable brush (Wiltek Medical, Winston-Salem, NC) was inserted into the working channel of the bronchoscope and advanced to the airways distal to the orifice of the desired lobar bronchus. Small airway epithelial samples were obtained from the 10th to 12th order bronchi by sliding the brush back and forth on the epithelium 10 to 20 times at 8 to 10 sites. For each brush, after withdrawing from the bronchoscope, the cells were dislodged from the brush by flicking the brush tip in 5 ml of ice-cold PneumaCult-Ex Plus Medium (StemCell Technologies, Cambridge, MA). The airway epithelial cells collected by brushing were pelleted by centrifugation (250×*g*, 5 min) and disaggregated by resuspension in 0.05% trypsin-ethylenediaminetetraacetic acid (EDTA) for 5 min, 37 °C. Trypsinization was stopped by addition of 4-(2-hydroxyethyl)-1-piperazineethanesulfonic acid buffered saline (HEPES) buffered saline, (Lonza, Basel, Switzerland) supplemented with 15% fetal bovine serum (FBS; Life Technologies, CA), and the cells were again pelleted at 250×*g*, 5 min. The pellet was resuspended with 5 ml of phosphate buffered saline, pH 7.4 (PBS), at 23 °C, then centrifuged at 250×*g*, 5 min. Following centrifugation, the cells (2.5 × 10^5^) were resuspended and plated in T25 flasks in 5 ml of PneumaCult-Ex Plus Medium and maintained in a humidified atmosphere of 5% CO_2_, 37 °C. The next day, unattached cells were removed by replacing the medium and thereafter, every 2 days. When the cells were 70% confluent, they were characterized by immunohistochemical staining of tryspinized cytopreps using cell type specific markers as being > 99% BC (KRT5^+^, TP63^+^, CD151^+^, β-tubulin IV^-^, MUC5AC^-^, TFF3^-^, CC10^-^, chromogranin A^-^ and N-cadherin^-^)^55^.To passage the cells, the primary BC were seeded at a cell density of 3000 cells/cm^2^ in PneumaCult-Ex Plus Medium. The following day, the medium was replaced with fresh medium every 2 days. To assess cytokine release, BCs (at passage 1) from HIV‾ and HIV^+^ nonsmokers were seeded in type IV collagen-coated 6-well plate at 2.5 × 10^5^/ml. Cells were incubated for 2 days and culture supernatants were collected for Western analyses.

### Culture of primary human airway basal cells

For the studies with normal, HIV‾ basal cells exposed in vitro to HIV, normal human airway basal cells (Lonza, Walkersville, MD) were seeded at 3000 cells/cm^2^ into plastic flasks and maintained in BEGM medium supplemented with 1% penicillin/streptomycin (Life Technologies, Grand Island, NY), 0.5% amphotericin B (Life Technologies) and 0.1% gentamicin (Sigma, St Louis, MO) in a 5% CO_2_, 37 °C humidified incubator with media replaced every 2 to 3 days. Once the cells reached 70% confluence they were harvested with 0.05% trypsin- EDTA (Life Technologies) for 5 min at 37 °C, with the reaction stopped by addition of HEPES supplemented with 15% fetal bovine serum (FBS, Life Technologies).

### Production of infectious HIV stocks

HIV-1 stocks (X4-tropic NL4-3 and R5-tropic AD8) were generated by calcium phosphate transfection of HEK293T cells (MBS mammalian transfection kit, Agilent Technologies, Santa Clara, CA) with the proviral vector, pNL-4-3, replication-defective pNL4-3-luc and pNLAD8 (NIH AIDS Research and Reference Reagent Program). Culture supernatants were collected after 48 h post-transfection and passed through 0.45 μm filters. Mock supernatants from non-transfected 293 T cells were collected for each preparation. Both mock and viral supernatants were prepared with the same number of 293 T cells and culture medium except no proviral plasmid in mock during transfection. Mock and viral supernatants were concentrated using LentiX reagent (Clontech, Mountain View, CA) and the viral pellet was resuspended in tissue-culture graded PBS after centrifugation. Quantification of the HIV p24 capsid protein was determined by HIV-1 p24 ELISA (Immuno Diagnostics, Woburn, MA) according to manufacturer’s instructions. The volume of viral supernatant added to the BC was in the range of 1:30 -1:60 dilution in BC culture (i.e., not more than 34 µl per ml of culture medium).

### Cytokine gene expression by TaqMan PCR

To assess the effect of HIV on basal cell cytokine gene expression, basal cells (5 × 10^4^) were exposed to increasing HIV input (p24 from 5 to 200 ng/ml in 300 μl) for 2 days. Heat-inactivated HIV was used as a control. Total RNA was extracted using Trizol reagent (Invitrogen) and the aqueous phase was purified using an RNAEasy MinElute RNA purification kit (Qiagen). RNA concentration was determined using a NanoDrop ND-100 spectrophotometer (NanoDrop Technologies). First-strand cDNA was synthesized from 0.5 µg of total RNA using TaqMan Reverse Transcription Reagents with random hexamer as primer (Applied Biosystems). All samples were analyzed in triplicate at cDNA dilution of 1:10. All reactions were run on an Applied Biosystems Sequence Detection System 7500 and relative expression levels determined using the dCt method with 18S ribosomal RNA as an endogenous control. The primers were obtained from Applied Biosystems, including IL-8 (Hs00174103_m1), GM-CSF (Hs00929873_m1), ICAM-1 (Hs00164932_m1), IL-1β (Hs00174097_m1) and 18s RNA (Hs99999901_s1). All primers were optimized for TaqMan PCR and referenced in recent studies^56–60^.

### Western analysis

BC (5 × 10^4^) were incubated with HIV or heat-inactivated HIV. Briefly, 25 µl supernatant from 5 × 10^4^ cells cultured for 2 days in 300 μl of BEGM were used for cytokine analysis. Culture supernatants were mixed with 5 × SDS PAGE loading buffer containing 50 mM dithiothreitol (DTT) added to each sample. The samples were then boiled for 5 min and analyzed using NuPAGE 4–12% Bis–Tris gradient gels (Invitrogen) and subsequently transferred onto Invitrolon PVDF membranes using XCell II Blot Module (Invitrogen). The membranes were then blocked at room temperature in PBS containing 0.1% Tween-20 (PBST) and 5% BSA for 1 h. After blocking, the immobilized proteins were reacted with the following antibodies in PBS containing 3% BSA at 4 °C with overnight shaking: IL-8 (1:1000; Abcam); GM-CSF (1:5000; Peprotech); ICAM-1 (1:500; Novus Biologicals); and IL-1β (1:2000; R & D Systems). Following the primary antibody incubation, membranes were washed three times for 5 min each with PBST, followed by incubation with an anti-rabbit, anti-goat or anti-mouse antibody conjugated to horseradish peroxidase (1:10,000; Santa Cruz Biotechnology) in PBS containing 3% BSA for 1 h at room temperature with shaking. The membranes were then washed again three times for 5 min with PBST and twice with PBS, and antibodies visualized after the addition of ECL Western analysis detection reagents (GE Healthcare Biosciences) by exposure to X-ray film.

### ELISA

To quantify IL-8 and GM-CSF levels released by BC, culture supernatants were harvested from basal cell cultures and spun at 10,000 rpm to remove cell debris. IL-8 and GM-CSF levels in the culture supernatants were measured using ELISA kits (RayBiotech, Norcross, GA), according to the manufacturer's instructions.

### Alveolar macrophage (AM) migration

Human alveolar macrophages from normal nonsmokers (n = 3) were obtained from bronchoalveolar lavage (BAL) from healthy nonsmokers as previously described^47^. BAL fluid was filtered through 2 layers of gauze and centrifuged at 1200 rpm for 5 min, 4 °C. Cells were washed twice in RPMI 1640 containing 10% FBS, 50 U/ml penicillin, 50 μg/ml streptomycin, and 2 mM glutamine (Invitrogen, Carlsbad, CA). Cells were suspended in complete medium, and cell viability was estimated by trypan blue exclusion and expressed as a percentage of the total cells recovered. Total cell number was determined by counting in a hemocytometer. Cells recovered from BAL fluid were seeded in 6-well plastic culture dishes (10^6^ per ml) and alveolar macrophages were purified by 24 h adherence at 37 °C in a 5% CO_2_ humidified incubator. Nonadherent cells were removed by thorough washing with RPMI 1640. AM represented ≥ 95% of cells^47,61–63^.

Chemotaxis was measured using CytoSelect 24-well cell migration assay kit (pore size: 5 μm, Cell Biolabs). Briefly, alveolar macrophages (3 × 10^5^ cells in 200 μl) in serum-free RPMI were plated in the upper compartment overnight. Cells were washed with sterile PBS twice and 200 μl of serum-free RPMI was added to the upper chamber. BC-conditioned media (200 μl) were diluted with serum-free RPMI in 1:1 (total volume: 400 μl) added to the lower compartment. For blocking experiments, BC-conditioned media was pre-incubated with anti-IL-8 neutralizing antibody (clone # 6217) and corresponding mouse IgG1 control (clone # 11,711; both at 2 μg/ml, endotoxin level < 0.10 EU per 1 μg of the antibody by the LAL method; R & D Systems) for 30 min at 37 ºC prior to addition to the lower chamber. The chemotactic chambers were incubated for 3 h at 37 ºC in humidified incubator with 5% CO_2_. Following termination of the assay, the bottom of polycarbonate filters were removed from the chambers and rinsed with 200 μl of sterile PBS to dislodge the migratory cells underneath the filter to the lower chamber. The numbers of macrophages that had migrated from the top to the bottom of the polycarbonate filter were counted by light microscopy.

### Neutrophil migration

Human neutrophils were isolated from peripheral blood (EasySep Direct Human Neutrophil Isolation Kit, StemCell Technologies, Cambridge, MA). Briefly, 5 ml of fresh blood collected in a K2EDTA tube was incubated with 250 μl of isolation cocktail and 250 μl of Rapid Sphere (StemCell Technologies) at 23 °C, 5 min. The sample was topped up to 12 ml by adding Mg^2+^ and Ca^2+^-free PBS containing 1 mM EDTA and mixed gently. The sample tube was placed in a Big Easy magnet (StemCell Technologies) and incubated for 5 min to allow unwanted cells attached to the magnet. The sample tube was inverted, and enriched cell suspension was poured into a new tube for another round of magnetic separation. A pure neutrophil cell suspension (CD16^+^ and CD66b^+^) was obtained after three rounds of separation and purity was > 95% as assessed by flow cytometer (BD Fortessa; see Supplemental Fig. 9).

Chemotaxis was measured using the CytoSelect 24-well cell migration assay kit (pore size, 3 μm, Cell Biolabs). Freshly isolated neutrophil (3 × 10^5^ cells in 200 μl per insert) in serum-free RPMI were plated in the upper compartment. BC-conditioned media (100 μl) were diluted with serum-free RPMI in 1:4 (total volume: 400 μl) added to the lower compartment. For blocking experiments, BC-conditioned media were pre-incubated with mouse IgG_1_ control and anti-IL-8 neutralizing antibody (both at 2 μg/ml, R & D Systems) for 30 min, 37ºC prior to addition to the lower chamber. The chemotactic chambers were incubated for 1 h, 37ºC in humidified incubator with 5% CO_2_. Following termination of the assay, the bottom of polycarbonate filters were removed from the chambers and rinsed with 200 μl of sterile PBS to dislodge the migratory cells underneath the filter to the lower chamber. The numbers of macrophages that had migrated from the top to the bottom of the polycarbonate filter were counted by light microscopy.

### Effect of BC on alveolar macrophage proliferation

Freshly isolated alveolar macrophages (5 × 10^4^) in serum-free RPMI were plated in 96 well for 2 h to allow cell attachment. Cells were washed with PBS twice and incubated with complete RPMI. To assess the effect of BC-released mediators on AM proliferation, BrdU Cell Proliferation ELISA kit was used (Abcam). Conditioned media from untreated, HIV-treated and heat-inactivated HIV-treated BC (20 μl) were diluted in complete RPMI in 1:5 (total volume: 100 μl) and added to AM. For blocking experiment, BC-conditioned media were pre-incubated with mouse IgG_1_ control and anti-GM-CSF neutralizing antibody (both at 10 μg/ml, R & D Systems) for 30 min at 37ºC prior to addition to AM culture. After 3 days of incubation, cells were pulsed with BrdU for 16 h. BrdU (a thymidine analog) was incorporated into the DNA of dividing cells. To detect incorporated BrdU, cells were fixed, permeabilized and incubated with detector anti-BrdU monoclonal antibody for 1 h. Unbound antibody was washed away and horseradish peroxidase-conjugated goat anti-mouse antibody was added which bound to the detector antibody. The horseradish peroxidase catalyzed the conversion of the chromogenic substrate tetra-methylbenzidine (TMB) from a colorless solution to a blue solution and turned yellow after the addition of stopping reagent. The intensity of colored product was proportional to the amount of BrdU incorporated in the cells. The colored reaction product is quantified using a spectrophotometer set at a dual wavelength of 450/550 nm. Each sample was run in triplicate.

### Statistical analysis

Unless otherwise mentioned, data were expressed as mean ± SD from three independent experiments. Statistical analysis was carried out and the data were subjected to statistical analysis using unpaired two-tailed Student’s t-tests with unequal variance. Values of p < 0.05 were considered significant.

## Results

### Small airway BC in HIV^+^, HAART-treated nonsmokers exhibit an inflammatory phenotype ex vivo

**Figure 1:**
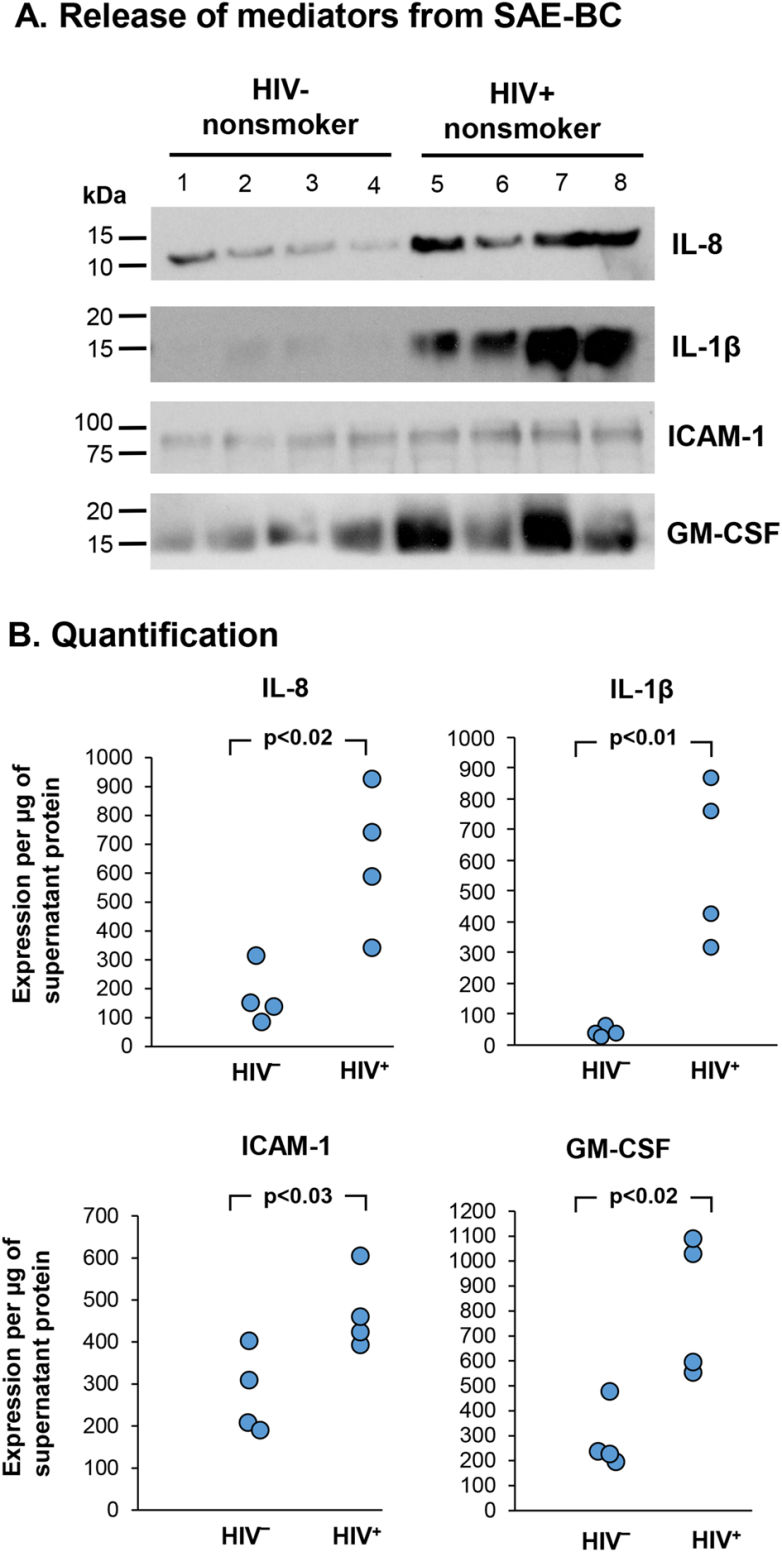
Small airway BC from HAART-treated HIV^+^ nonsmokers are induced to release inflammatory mediators in vivo. Small airway BC (5 × 10^5^ in 1 ml) from 4 HIV^-^ and 4 HIV^+^ nonsmokers (all with viral load < 20 copies/ml) were plated onto type IV collage-coated 6-wells and incubated for 2 days. Culture supernatants were collected for assessment of cytokine release. (**A)** Western analyses of IL-8, IL-1β, GM-CSF and ICAM-1 from HIV^-^ (lanes 1–4) and HIV^+^ nonsmokers (lanes 5–7). The original images of full-length immunoblot for each cytokine were shown in Supplemental Fig. 1. (**B)** Quantification of IL-8, IL-1β, GM-CSF and ICAM-1 release by small airway BC. Western analysis from HIV‾ and HIV^+^ small airway BC (n = 4 for each group) were quantified by ImageJ software and normalized with the total amount of supernatant protein as densitometry (pixel intensity) per µg of supernatant protein.

Given that lung inflammation and airway dysfunction is observed in HIV^+^, HAART-treated individuals^11,20,36,37,64–69^, we hypothesized that BC isolated from HIV^+^ nonsmokers had been modulated in vivo to adapt an inflammatory phenotype. A cohort of age matched healthy nonsmokers (n = 4) and HAART-treated HIV^+^ nonsmokers (n = 4) was used in this study (Table 1). Small airway BC from HIV‾ and HAART-treated HIV^+^ nonsmokers were isolated were plated on type IV collagen–coated 6-well and conditioned media were collected for cytokine assessment after 2 days. Western analysis showed that BC from HIV^+^ nonsmokers had been induced to increase released of IL-1 β, IL-8, GM-CSF and ICAM-1 (lanes 5–8) *vs* healthy controls (lanes 1–4; ICAM-1, p < 0.03; IL-8, p < 0.02; IL-1 β, p < 0.01; GM-CSF, p < 0.02, Figs. 1A,B). These findings provide evidence that small airway BC from HIV^+^ nonsmokers are induced in vivo to adapt an inflammatory phenotype.

### HIV induces human airway BC to express and release inflammatory mediators

**Figure 2:**
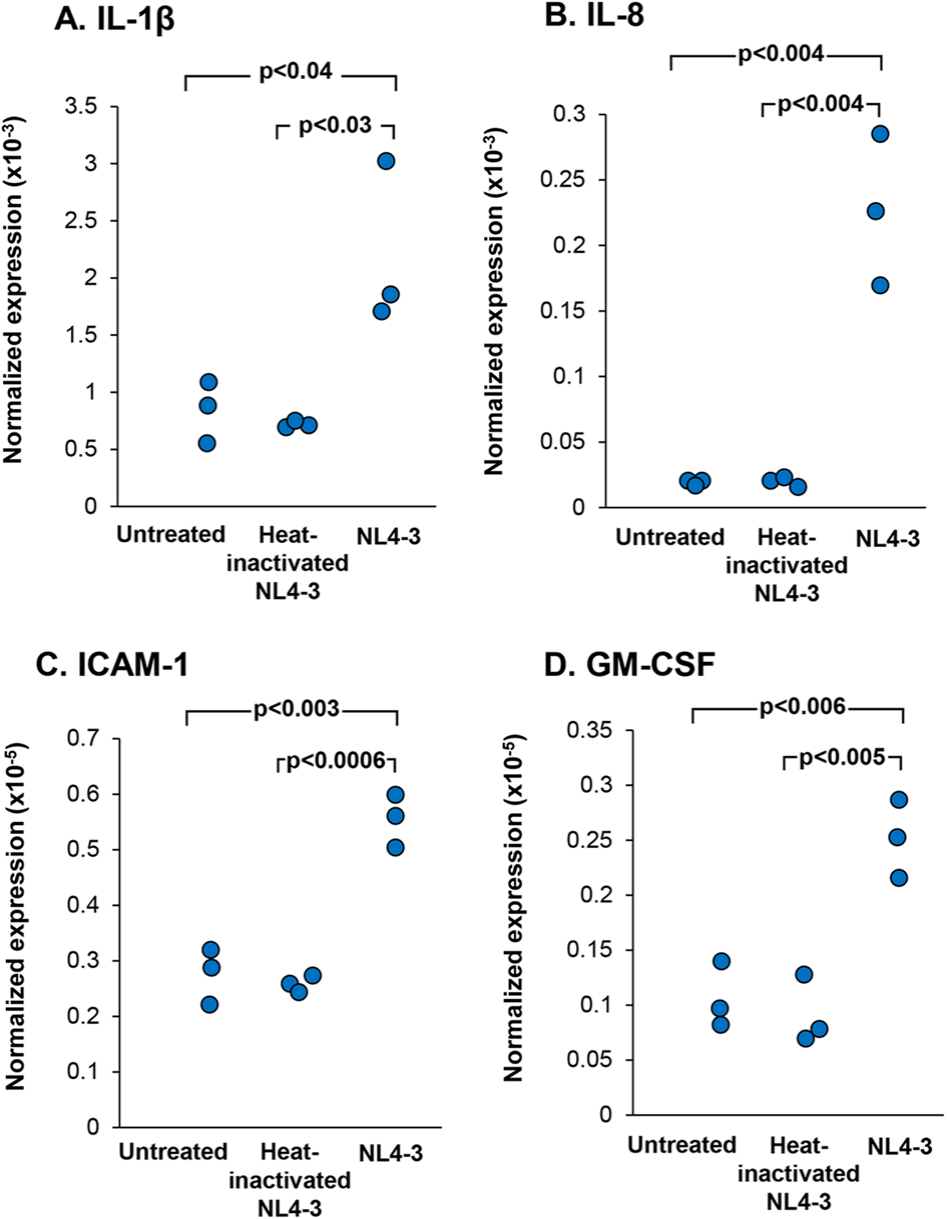
HIV induced cytokine gene expression by airway BC. BC were exposed to HIV or heat-inactivated HIV for 2 days. TaqMan quantitative PCR analysis was performed to assess cytokine gene expression shown in (**A)**, IL-1β. (**B)**, IL-8; (**C)**, ICAM-1; and (**D)**, GM-CSF. The data were normalized to 18s RNA. Results shown are the data from three independent experiments.

With the knowledge that the lung remains a reservoir of HIV^+^ in HAART-treated individuals despite low blood HIV levels^20–23,25,26,28^, we hypothesized that HIV may interact with BC and induce the BC to release inflammatory mediators. We have previously shown that some HIV bind to BC, activating the BC through heparan sulfate proteoglycan receptors, but HIV does not replicate in normal human airway BC^46^. HIV binding to the BC initiates a cascade of events mediated through MAPK/ERK signaling pathways and induces increased expression and secretion of matrix metalloproteases (MMP), including MMP-9, capable of inducing tissue destruction^46^. To evaluate the hypothesis that HIV induces normal BC to acquire an inflammatory phenotype that may contribute to this inflammatory process, BC were exposed to increasing HIV_NL4-3_ (p24 from 5 to 200 ng/ml) for 48 h in vitro. Total RNA and culture supernatants were analyzed for cytokine gene expression and release. We focused on GM-CSF, IL-8, IL-1β and ICAM-1 as these mediators are relevant to COPD pathogenesis and are found at increased levels in epithelial lining fluid from HIV^+^ individuals^35,63^ and HIV^+^ BC-conditioned media when cells were cultured ex vivo (Fig. 1A). TaqMan quantitative PCR showed HIV induced upregulation of GM-CSF, IL-8 and IL-1β in a dose-dependent fashion (p < 0.05 as compared to no viral input; Supplemental Fig. 3). BC were also treated with HIV or heat-inactivated HIV for 48 h. When compared to untreated and heat-inactivated HIV, HIV significantly induced expression of IL-1β (Fig. 2A), IL-8 (Fig. 2B), ICAM-1 (Fig. 2C) and GM-CSF (Fig. 2D) (all p < 0.05; Fig. 2). Heat-inactivated HIV had no significant effect on inflammatory cytokine gene expression. To ensure the HIV-induced inflammation, mock and replicative defective HIV were included in our study. BC were treated with the same volume of mock control and HIV_NL4-3_ (i.e., 23 µl) for 48 h. Expression of inflammatory cytokine genes were assessed in both treatment groups. HIV induces upregulation of GM-CSF, IL-8, IL-1β and ICAM-1 as compared to mock control (all p < 0.02; Supplemental Fig. 4). We also measured LDH cytotoxicity in mock-treated BC and HIV-treated BC after 72 h. Both mock and HIV did not affect BC viability as compared to untreated BC (all < 5% of LDH cytotoxicity when compared to whole cell lysate (positive control; Supplemental Fig. 5). 293 T-derived replication-defective HIV NL4-3-luc was produced and concentrated using Lenti-X concentrator. BC were treated with 293 T-derived HIV NL4-3-luc for 48 h and assessed for expression of inflammatory cytokine genes by TaqMan PCR. The effect of HIV NL4-3-luc on expression of inflammatory cytokine genes was insignificant compared to untreated BC (Supplemental Fig. 6).

**Figure 3:**
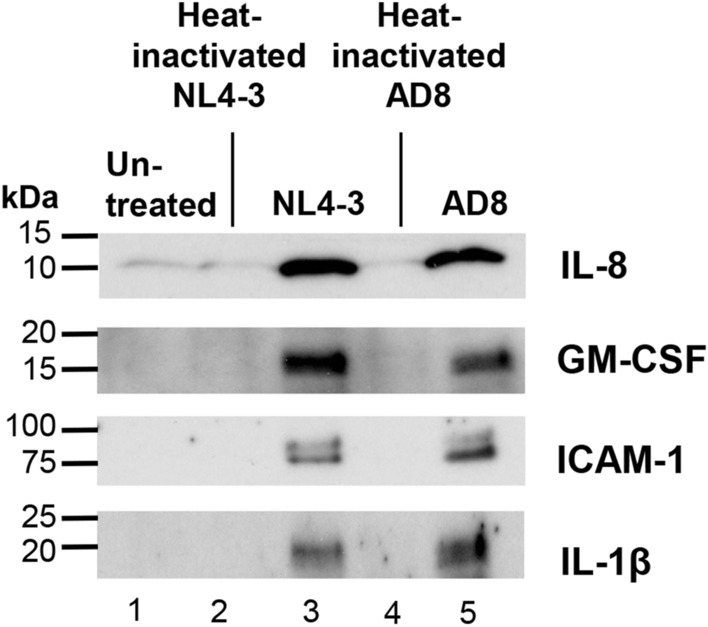
Western analysis of HIV-induced cytokine secretion by airway BC. BC were exposed to HIV or heat-inactivated HIV for 2 days. Culture supernatants were collected for assessment of cytokine release. Western analyses of IL-8, GM-CSF, IL-1β and ICAM-1 are shown. Lane 1—untreated BC; lane 2—heat-inactivated NL4-3-treated BC; lane 3—NL4-3-treated BC; lane 4—heat-inactivated AD8-treated BC; lane 5—AD8-treated BC. The original images of full-length immunoblot for each cytokine was shown in Supplemental Fig. 2.

The corresponding cytokine levels were evaluated in BC-conditioned media by Western analyses (Fig. 3). Secreted and activated form of IL-8 and IL-1b were quantified in this study. Consistent with the TaqMan gene expression data, there were increased levels of IL-1β, IL-8, ICAM-1 and GM-CSF in HIV (both NL4-3 and AD8, lanes 3 and 5; Fig. 3) when compared to untreated and heat–inactivated HIV-treated BC respectively (lanes 1, 2 and 4; Fig. 3).

### HIV induces BC to release mediators that induce migration of AM and neutrophils

**Figure 4:**
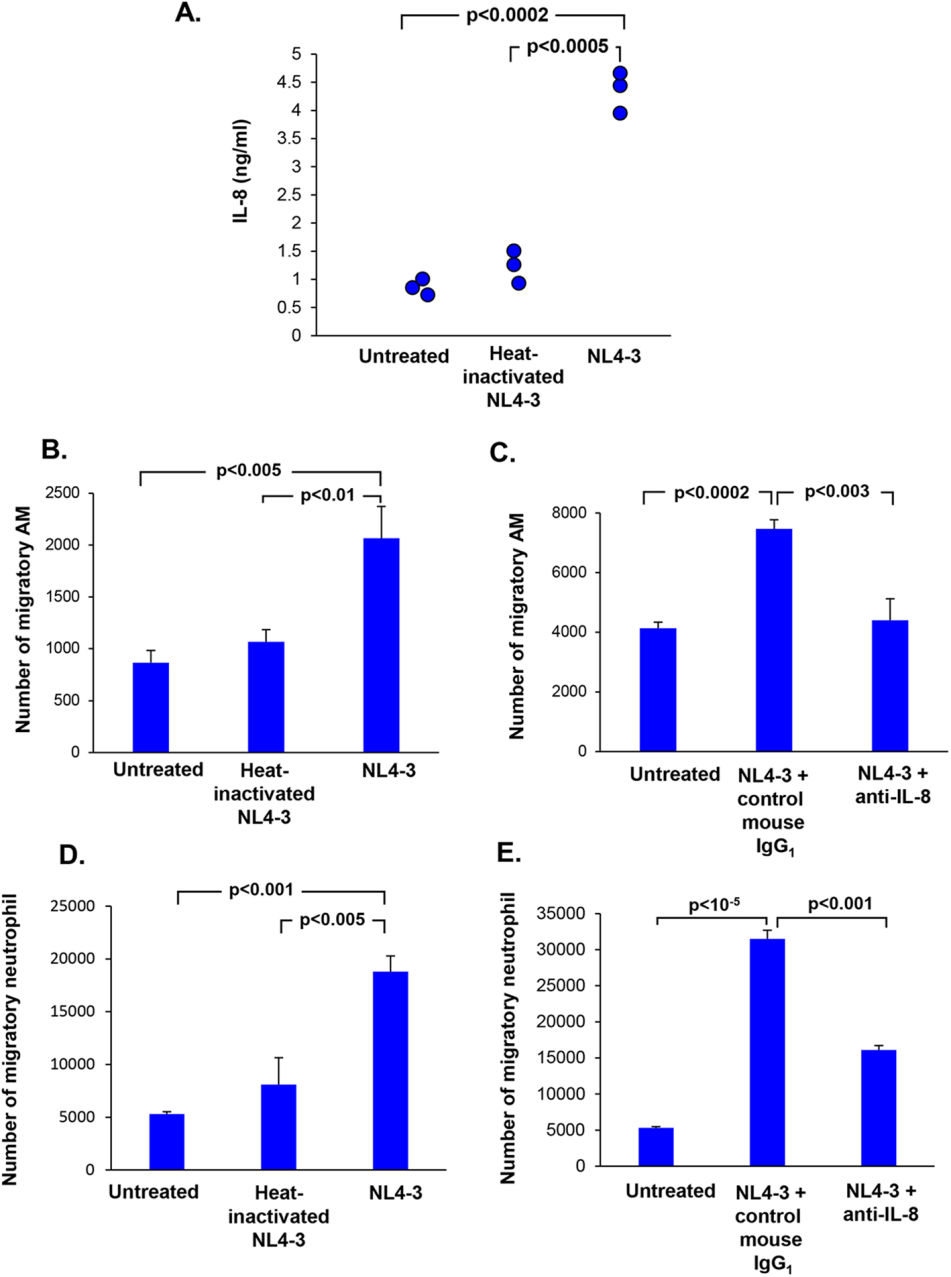
HIV-induced BC expression of IL-8 mediates AM and neutrophil migration. BC were exposed to HIV or heat-inactivated HIV for 2 days. (**A**) Quantification of IL-8 in culture supernatants of untreated, heat-inactivated HIV and HIV- treated BC cultures by ELISA. (**B**) HIV-induced BC release of these mediators induce migration of human alveolar macrophages and neutrophils. Alveolar macrophages obtained from an HIV negative normal subject were plated in serum-free medium in the upper chamber and incubated with BC-conditioned media from untreated, NL4-3 treated and heat-inactivated NL4-3-treated BC culture in the lower chamber of transwell system. The number of migratory cells were quantified after 3 h. (**C**) Migration of human alveolar macrophages mediated by BC-release of IL-8. BC-conditioned media were pre-treated with mouse IgG1 control or anti-IL-8 neutralizing antibody (both at 2 µg/ml) for 30 min prior to addition to the lower chamber. Human alveolar macrophages were plated on the upper chamber and incubated with conditioned media from untreated BC, HIV-BC pretreated with mouse IgG_1_-treated or HIV-BC pretreated with anti-IL-8 neutralizing antibody. The number of migratory alveolar macrophages were counted after 3 h. Data shown are the mean ± SD of one representative of three independent experiments performed in triplicate. (**D**) Stimulation of migration of neutrophils. Human neutrophils in serum-free RPMI were plated in the upper chamber and incubated with BC-conditioned media from untreated, HIV-treated and heat-inactivated HIV-treated BC culture in the lower chamber of transwell system. The number of migratory cells were quantified after 1 h of incubation. (**E**) Migration of human neutrophils mediated by BC-released IL-8 in conditioned media. BC-conditioned media were pre-treated with mouse IgG_1_ control and anti-IL-8 neutralizing antibody (both at 2 µg/ml) for 30 min prior to addition to the lower chamber. Human neutrophils were plated on the upper chamber and incubated with conditioned media from untreated BC, HIV-BC pretreated with mouse IgG_1_-treated, HIV-BC pretreated with anti-IL-8 neutralizing antibody or HIV-treated BC. The number of migratory alveolar macrophages were counted after 1 h of incubation. Data are presented as mean ± SD of one representative of three independent experiments performed in triplicate.

We next examined whether the mediators released by BC could functionally influence other cells in the lung such as AM and neutrophils, cells that are important components in the pathogenesis of emphysema/COPD^70,71^. Consistent with the gene expression data, there was an increased secretion of IL-8 from HIV-exposed BC *vs* untreated (4.35 *vs* 0.86 ng/ml, p < 0.0002) and *vs* heat-inactivated HIV (4.35 *vs* 1.32 ng/ml, p < 0.0005) as confirmed by ELISA (Fig. 4A). Heat-inactivated HIV did not have any effects on IL-8 release (Fig. 4A).

To assess if HIV-BC released mediators could attract inflammatory cells, AM and neutrophil migration assays were carried out using HIV-BC conditioned media. Human AM from normal healthy nonsmokers were plated on the upper chamber of transwell and incubated with conditioned media from untreated BC, heat-inactivated HIV-treated BC and HIV-treated BC in the lower chamber, and the number of migratory cells were quantified after 3 h of incubation. There was an increase in the number of migratory AMs in HIV-treated BC as compared to untreated control (p < 0.005; Fig. 4B) and heat-inactivated HIV (p < 0.01; Fig. 4B).

Chemoattractant studies with normal AM and normal neutrophils demonstrated that the IL-8 released by BC exposed to HIV was functional, capable of attracting normal human (Fig. 4B) and neutrophils (Fig. 4D). With the restriction of the COVID-19 pandemic, we had difficulties to obtain fresh AM from one individual to set up all treatment groups in a single experiment. Using cells from other individuals, pretreatment of HIV-treated BC-conditioned media with anti-IL-8 antibody significantly blocked migration of AM when compared to pretreatment of HIV-treated BC-conditioned media with the mouse IgG_1_ control (p < 0.003; Fig. 4C). Similar to AM, there was an increased number of migratory neutrophils in HIV-treated BC conditioned medium as compared to untreated BC (p < 0.001; Fig. 4D) and compared to heat-inactivated HIV-BC (p < 0.005; Fig. 4D). In separate experiments using cells from different nonsmokers, pretreatment of HIV-BC conditioned media with anti-IL-8 significantly inhibited neutrophil migration as compared to HIV-BC conditioned media pretreated with mouse IgG_1_ control (p < 0.001; Fig. 4E). Same findings were observed from a complete experiment including all the controls and treatment groups using neutrophils from one individual (Supplemental Fig. 7). In addition, we assessed neutrophil migration using conditioned medium from BC of HIV‾ and HIV^+^ nonsmoker cultured ex vivo. Compared to HIV‾ nonsmoker BC, HIV^+^ BC released chemotactic factors which increased neutrophil migration, suggesting that HIV^+^ BC exhibited inflammatory phenotypes (p < 0.0005; Supplemental Fig. 8).

### HIV-induced BC release mediators that stimulate AM proliferation

**Figure 5:**
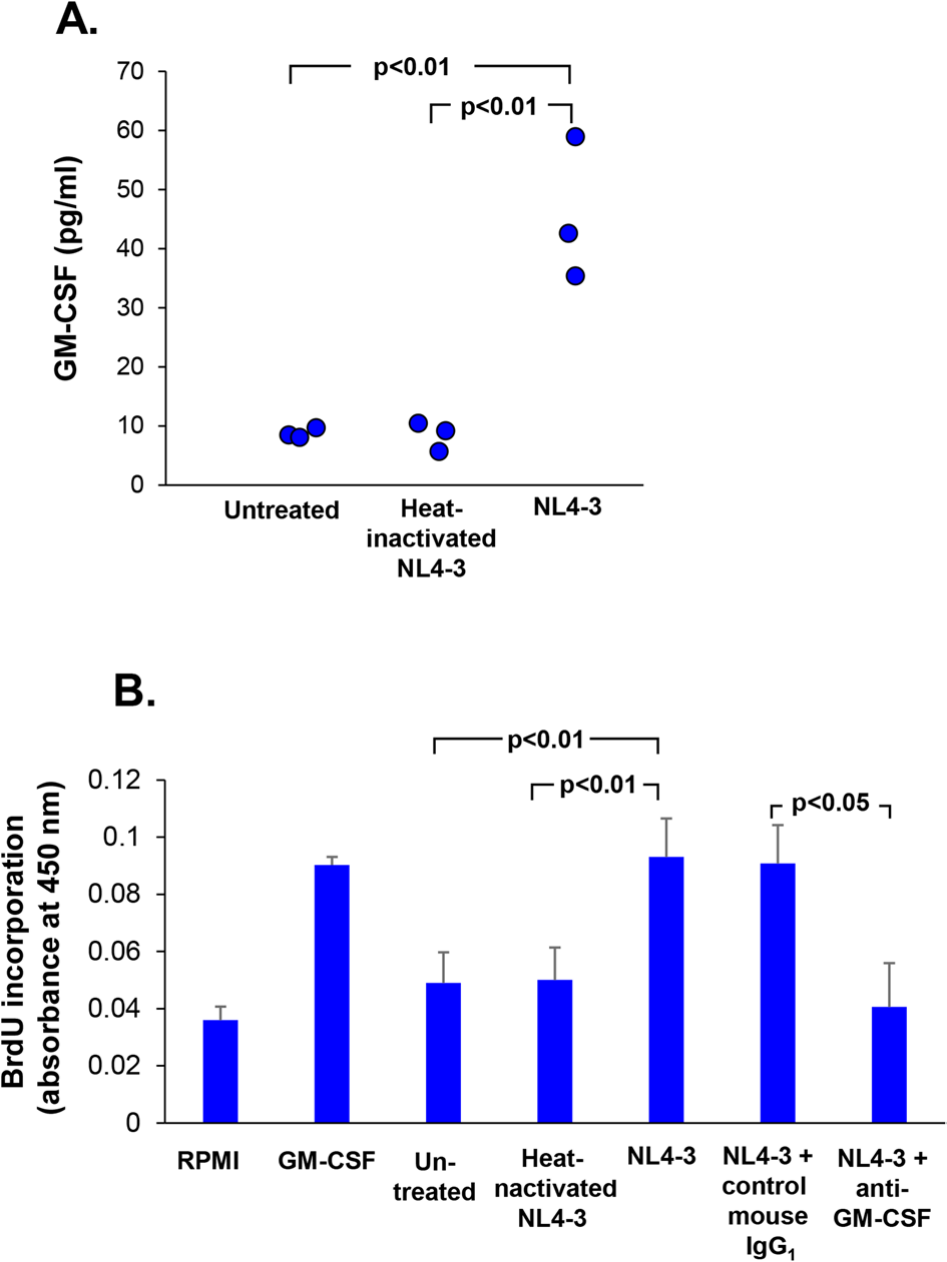
HIV-treated BC release GM-CSF that stimulates AM proliferation. (**A)** Quantification of GM-CSF in BC-conditioned medium by ELISA. (**B)** Proliferation of human AM is partially mediated by BC-released GM-CSF in conditioned media. Alveolar macrophages in serum-free RPMI were plated in 96-well and incubated with BC-conditioned media from untreated, HIV-treated and heat-inactivated HIV-treated BC culture. RPMI and recombinant GM-CSF (20 ng/ml) serve as background and positive controls, respectively. BC-conditioned media were pre-treated with mouse IgG_1_ control and anti-GM-CSF neutralizing antibody (both at 10 µg/ml) for 30 min prior to addition to the cells. Cell proliferation was measured by BrdU incorporation assay after 3 days of culture. Data represent the mean ± SD of one representative experiment (n = 3) performed in triplicate.

Based on the cytokine array and Western analyses, there was an increased release of GM-CSF level in HIV-reprogrammed BC. This was confirmed by ELISA, with increased GM-CSF in the supernatants of HIV-exposed BC *vs* untreated (9 *vs* 45 pg/ml; p < 0.01; Fig. 5A) and *vs* heat-inactivated HIV (8.4 *vs* 45 pg/ml; p < 0.01; Fig. 5A). BC exposed to heat-inactivated HIV did not have any increased GM-CSF in the supernatant (Fig. 5A).

To examine if GM-CSF released by HIV-BC could stimulate AM proliferation, freshly isolated AM were exposed to BC-conditioned media from untreated, heat-inactivated HIV and HIV-BC for 3 days. Assessment using BrdU incorporation revealed that HIV-BC conditioned stimulated AM proliferation compared to untreated (p < 0.01; Fig. 5B) and *vs* heat-inactivated HIV (p < 0.01; Fig. 5B). Pretreatment of HIV-BC conditioned media with anti-GM-CSF significantly inhibited AM proliferation compared to mouse IgG_1_ control (p < 0.04; Fig. 5B).

## Discussion

With the advent of effective anti-retroviral therapy, the mortality rate of individuals infected with HIV has been significantly declined^2^. However, the increased life span within the HIV^+^ population has been accompanied by an increased prevalence of aging-related chronic disorders, including COPD^3,4,6–8,11,12,72^. The mechanisms of HIV-associated COPD is not fully understood but is clearly associated with chronic lung inflammation, dominated by activated AM, CD8^+^ T cells and neutrophils^13–15,30^. A variety of studies have shown the decline in lung function and pulmonary/airway abnormalities observed in HIV^+^ individuals despite effective HAART treatment^64,66–69,73,74^. Previous reports have shown that the lung is a reservoir for HIV despite systemic viral suppression with anti-retroviral therapy^20–23,25,26,30,31^. Given our recent observation that HIV can bind to heparan sulfate receptors on airway BC, resulting in reprogramming of the BC to up-regulate expression and release of MMP-9^46^, we hypothesized that HIV binding to BC may also induce the BC to an inflammatory phenotype that contributes to the chronic AM/neutrophil lung inflammation that characterizes HIV infection despite anti-retroviral therapy. Our findings demonstrated that small airway BC isolated from HAART-treated HIV^+^ nonsmokers release inflammatory mediators IL-8 IL-1β, ICAM-1 and GM-CSF. Consistent with the ex vivo data, HIV upregulates expression of these inflammatory mediators in normal airway BC in vitro. These HIV-BC derived mediators mediate migration of AM and neutrophils, and proliferation of alveolar macrophages in vitro.

### The lung in HIV infection

The lung serves as a HIV reservoir in both untreated and HAART-treated individuals^22–24,75^. Despite systemic viral suppression, HAART does not eliminate HIV reservoirs in compartments such as lung, lymph nodes, colon and the genital tracts^75^. Immunohistochemical study showed that gp120 immunoreactivity was observed in both HIV-infected lungs after HAART treatment^28^. Based on these studies, it appears that HIV-1 may compartmentalize to the area within the lung where HAART is not as effective. In the lung, AM are a major target for HIV-1^20^. HIV preferentially infects AM and impairs AM phagocytic activity^76^. Several studies have been shown that HIV-infected AM release oxidants and various cytokines to trigger immune activation, neutrophil infiltration, and express proteases such as MMPs for degradation of extracellular matrix^20,47,77^. It is conceivable that residual HIV in the lung might infect AM which serve as viral reservoirs to productively replicate HIV in the lung. Newly synthesized viral particles interact with neighboring HIV receptor-expressing cells such as epithelial cells, dendritic cells and lymphocytes. HIV binding to surface heparan sulfate expressed in airway basal cells reprograms BC to acquire destructive phenotypes and induces MMP-9 upregulation through MAPK signaling pathway^46^. The amount of viral load in the lung compartment of HAART-treated HIV individuals has not been well studied and quantified. In this study, BC were exposed to increasing HIV input (p24 from 5 to 200 ng/ml). HIV upregulates the expression of inflammatory genes in a dose-dependent manner (Supplemental Fig. 3). More importantly, BC isolated from HAART-treated HIV^+^ nonsmokers showed increased secretion of inflammatory mediators ex vivo when compared to HIV^-^ nonsmokers (Fig. 1).

There are increased numbers of AM, T lymphocytes, NK and neutrophils in the epithelial lining fluid of HIV^+^ individuals^30,31,78,79^. Prior studies have demonstrated that HIV infection leads to activation of alveolar macrophages to release pro-inflammatory cytokines and chemokines, and alters the inflammatory cell composition in the lower respiratory tract, including increased macrophages, neutrophils and lymphocytes^13–15,30,34,35,40,80–82^. Alveolar macrophages of HIV-infected individuals secrete increased levels of cytokines including IL-1, IL-8 and GM-CSF^30,40,80,82–85^. These cytokines play a role in triggering inflammatory responses, recruitment of immune cells and promote survival/proliferation of inflammatory cells in the lung.

Our findings demonstrated that HIV-treated BC released mediators that induced AM and neutrophil migration. Ex vivo BC from HAART-treated HIV nonsmokers also released chemotactic factors, for example, IL-8 that induced neutrophil migration. Relevant to COPD pathogenesis, the numbers of both AM and neutrophils significantly increased in sputum and BAL of COPD patients^86–88^. Both AM and neutrophils play an important role in airway inflammation. They secrete various serine proteases, including matrix metalloproteinases, proteinase 3, cathepsins and neutrophil elastase which cause mucus hypersecretion and alveolar destruction^70,71,89–92^.

### Airway epithelium as “inflammatory cells”

While HIV-infected AM play a role in mediating lung inflammation associated with HIV infection, little attention has been focused on a role of the airway epithelium in the pathogenesis of HIV associated lung inflammation. It is known that HIV can disrupt lung epithelial integrity^38^, suppresses tracheobronchial mucociliary clearance^93^ and that the HIV-1 envelope protein gp120 stimulates mucus formation in bronchial epithelial cells via the CXCR4/α7-nicotinic acetylcholine receptor pathway^28^.

The present study adds to the concept that airway epithelium BC contribute to the initiation and maintenance of the chronic lung inflammation associated with HIV infection by HIV binding to, and modulating the BC to adapt an inflammatory phenotype that includes increased expression of IL-1β, IL-8, ICAM-1 and GM-CSF. This is consistent with prior studies demonstrating that human differentiated airway epithelial cells have the capacity to contribute to airway inflammation^70,94–97^. Airway epithelial cells are capable of releasing a variety of pro-inflammatory mediators including chemokines, cytokines and growth factors through various signaling pathways in response to cigarette smoke and other pathogens such as respiratory syncytial virus and influenza virus^70,95–97^. These mediators play an important role in the pathogenesis of COPD by orchestrating inflammation through recruitment, activation and survival of inflammatory cells^70^.

There is extensive data demonstrating that HIV infection is associated with inflammation in the lung^11,20,33,35–37,42,98^. Compared to HIV‾ nonsmokers, analysis of lung epithelial lining fluid showed that HIV^+^ nonsmokers has demonstrated increased levels of a variety of inflammatory mediators, including GM-CSF, IFN-γ, IL-5, IL-6, IL-13, IL-8, MCP-1, CCL4, RANTES, I-TAC, I-309, IL-23, IL-17, PAI-1 and CD40 compared to HIV‾ nonsmokers^63^. Similarly, another study reported that chronic untreated HIV infection dysregulated the lung cytokine microenvironment and was incompletely restored by long-term ART^99^. Analysis of bronchoalveolar lavage fluid cytokine levels showed a significant increase in IL-8, IL-1β and IL-6 in asymptomatic HIV-infected ART-naive and ART-treated HIV^+^ individuals as compared to healthy individuals^99^. Increased levels of IL-8 have been found in bronchoalveolar lavage from HIV-infected nonsmokers and HIV^+^ subjects with *P. carinii* pneumonia^35,63,85^. AM from HIV^+^ individuals spontaneously release more GM-CSF and IL-8 than those from normal controls^30,80^, and there is an increased secretion of IL-1β from AM of HIV^+^ individuals as compared to normals^82,100,101^. Similar to our observations, X4 tropic HIV enhance the expression of ICAM-1 in in vitro differentiated airway epithelium^38^. ICAM-1 mediates interaction between polymorphonuclear leukocytes and airway epithelial cells and transmigration of neutrophils and macrophages in the lung^102^. In this case, HIV-induced ICAM-1 promotes immune cell infiltration, activation and local inflammation/damage in the lung. In addition to airway BC, it deserves further investigation that other mature airway differentiated cells including ciliated, mucus and club cells may also contribute to inflammation after HIV exposure.

Several mechanisms of HIV-associated lung diseases have been proposed including oxidative stress, imbalance of proteases/anti-proteases, direct effect of HIV or HIV-related proteins, use of HAART and opportunistic infections^7,11,37^. Using BC isolated from HAART-treated HIV^+^ nonsmokers in this study has limitations to precisely demonstrate if increased inflammatory cytokine expression is solely from HIV interaction with BC. It remains to be determined if other factors such as other HIV proteins, antiviral drugs and opportunistic infections may also contribute to lung inflammation in HIV^+^ nonsmokers. Further studies will be required to better understand the mechanisms of HIV-associated lung disorders.

Taken together, we have identified a novel, unexpected mechanism that HIV induces BC to adopt an “inflammatory phenotypes”, releasing mediators that attract and activate alveolar macrophages and neutrophils. These findings explain how lung inflammation in HIV^+^ individuals are generated and perpetuated and why there is an increased incidence of COPD in HIV^+^ individuals in the era of HAART.

## Supplementary Information

Supplementary Information.
