# Supplementary material for: HIV induces airway basal progenitor cells to adopt an inflammatory phenotype
Source: Sci Rep. 2021 Feb 17;11:3988. doi: 10.1038/s41598-021-82143-1 (PMC7889866; doi:10.1038/s41598-021-82143-1)

Supplemental Data Cover Page

HIV Induces Airway Basal Progenitor Cells to Adopt an Inflammatory Phenotype

Nancy P.Y. Chung[1], K.M. Faisal Khan[1], Robert J. Kaner[1,2], Sarah L. O'Beirne[1,2] and Ronald G. Crystal[1,2]

[1]Department of Genetic Medicine

and

[2]Department of Medicine
Weill Cornell Medical College
New York, New York

Supplemental Experimental Procedures

Below is a summary of the inclusion/exclusion criteria for HIV- and HIV+ nonsmokers enrolled in this study:

Healthy HIV^{-} nonsmokers

Inclusion criteria

- Males and females, age 18 or older
- Must be capable of providing informed consent
- Self-reported never-smokers, with current smoking status validated by the absence of nicotine metabolites in urine (nicotine <2 ng/ml and cotinine <5 ng/ml)
- Normal physical examination
- Good overall health without history of chronic lung disease, including asthma, and without recurrent or recent (within 3 months) acute pulmonary disease
- Normal routine laboratory evaluation, including general hematologic studies, general serologic/immunologic studies, general biochemical analyses, and urine analysis
- Not taking any medications relevant to lung disease or having an effect on the airway epithelium
- Negative HIV serology
- Normal chest X-ray (PA and lateral) and chest HRCT
- Normal electrocardiogram (sinus bradycardia, premature atrial contractions are permissible)
- Females - not pregnant
- No history of allergies to medications to be used in the bronchoscopy procedure
- Willingness to participate in the study

Exclusion criteria

- Unable to meet the inclusion criteria
- Current active infection or acute illness of any kind
- Evidence of malignancy within the past 5 years
- Current alcohol or drug abuse
- Pregnancy

Healthy HIV^{+} nonsmokers

Inclusion criteria

- Males and females, age 18 or older
- Must be capable of providing informed consent
- Self-reported never-smokers, with current smoking status validated by the absence of nicotine metabolites in urine (nicotine <2 ng/ml and cotinine <5 ng/ml)
- Normal physical examination • Good overall health without history of chronic lung disease, including asthma, and without recurrent or recent (within 3 months) acute pulmonary disease
- Normal chest X-ray (PA and lateral) and chest HRCT • Normal electrocardiogram (sinus bradycardia, premature atrial contractions are permissible)

- Females - not pregnant
- No history of allergies to medications to be used in the bronchoscopy procedure
- Willingness to participate in the study
- Documented HIV infection (positive HIV 1 antibody ELISA with Western analysis confirmation)

Exclusion criteria

- Unable to meet the inclusion criteria
- Individuals in whom participation in the study would compromise the normal care and expected progression of their disease
- Evidence of malignancy within the past 5 years
- Current alcohol or drug abuse
- Pregnancy

LDH Cytotoxicity Assay

To measure the effect of HIV on BC survival, LDH cytotoxicity assay was used (Thermo Scientific). BC (1 $\times 10^4$/100 µl) were plated in 96 well and incubated overnight. On the next day, cells were treated with increasing does of HIV(p24 from 5 to 100 ng/ml) for 72 hours. Each treatment group was run in triplicate. Spontaneous LDH Activity Controls and Maximum LDH Activity Controls were included according to manufacturer's protocol. After 72 hours of incubation, 10 µl of water and lysis buffer were added to Spontaneous LDH Activity Controls (water) and Maximum LDH Activity Controls (10× Lysis Buffer) respectively and incubated at 37°C for 45 min. After that, 50 µL of each sample media (i.e. media from HIV-treated BC, mock-treated BC, Spontaneous LDH Activity Controls and Maximum LDH Activity Controls) were transferred to a 96-well flat-bottom plate and mixed with Reaction Mixture. After 30 min incubation at room temperature incubation, reactions were stopped by adding Stop Solution. Absorbance at 490 nm and 680 nm was measured using spectrophotometer to determine LDH activity. The percentage of cytotoxicity was calculated using this formula:

[HIV (or mock)-treated LDH activity – Spontaneous LDH activity] /

[Maximum LDH activity – Spontaneous LDH activity] x 100

Supplemental Figure Legends

Supplemental Figure 1. Small airway BC from HAART-treated HIV^+ nonsmokers are reprogrammed to spontaneously release inflammatory mediators *in vivo*. Small airway BC from 4 HIV^- and 4 HIV^+ nonsmokers (all with viral load <20 copies/ml) were plated onto type IV collage-coated 6-wells and incubated for 2 days. Culture supernatants were collected for assessment of cytokine release. The original images of full-length immunoblot for IL-8, IL-1β, GM-CSF and ICAM-1 from HIV^- (lanes 1-4) and HIV^+ nonsmokers (lanes 5-7) were shown. The lanes used for making Figure 1 were boxed in red.

Supplemental Figure 2. Western analysis of HIV-induced cytokine secretion by airway BC. BC were exposed to HIV or heat-inactivated HIV for 2 days. Culture supernatants were collected for assessment of cytokine release. Western analyses of IL-8, GM-CSF, IL-1β and ICAM-1 are shown. Lane 1- untreated BC; lane 2 – heat-inactivated NL4-3-treated BC; lane 3 – NL4-3-treated BC; lane 4 –heat-inactivated AD8-treated BC; lane 5 – AD8-treated BC. The original images of full-length immunoblot for each cytokine were shown. The lanes used for making Figure 3 were boxed in red.

Supplemental Figure 3. HIV induces expression of inflammatory mediators in dose-dependent manner. BCs were exposed to increasing viral input (p24 from 5 to 200 ng/ml) for 2 days, and the expression of inflammatory markers was quantified by Taqman PCR. The data were normalized to 18s RNA.

Supplemental Figure 4. Expression of inflammatory mediators in mock and HIV-treated BC. Cells were treated with HIV (p24 at 200 ng/ml) for 48 hours. Mock (same volume of supernatant from non-transfected 293T cells) was added and served as control in this experiment. Expression of inflammatory markers was quantified by Taqman PCR. The data were normalized to 18s RNA.

Supplemental Figure 5. Cell viability of mock and HIV-treated BC by LDH cytotoxicity assay. Cells were treated with HIV (p24 at 5 - 200 ng/ml) for 72 hr. Mock (same volume of supernatant from non-transfected 293T cells) was added and served as control in this experiment. Data represent the mean± SD of one representative experiment performed in triplicate.

Supplemental Figure 6. Expression of inflammatory mediators in untreated and HIV-luc-treated BC. Cells were treated with replication-defective HIV-luc (p24 at 200 ng/ml) for 48 hr. Expression of inflammatory markers was quantified by Taqman PCR. The data were normalized to 18s RNA.

Supplemental Figure 7. Migration of human neutrophils mediated by BC-released IL-8 in conditioned media. BC-conditioned media were pre-treated with mouse IgG_1 control and anti-IL-8 neutralizing antibody (both at 2 μg/ml) for 30 min prior to addition to the lower chamber. Human neutrophils were plated on the upper chamber and incubated with conditioned media from untreated BC, HIV-BC pretreated with mouse IgG_1-treated, HIV-BC pretreated with anti-IL-8 neutralizing antibody or HIV-treated BC. The number of migratory neutrophils were counted after 1 hr of incubation.

Supplemental Figure 8. HIV^+ BC released mediators that induced neutrophil migration. BC (2.5×10^5 cells/ml) from HIV^- and HIV^+ nonsmokers were plated on collagen-coated culture well and BC-conditioned media were collected after 48 hours. BC-conditioned media were diluted with serum-free RPMI at 1:4 dilution and added in the lower chamber. Human neutrophils were plated on the upper chamber of transwells and incubated with diluted conditioned media from HIV^- and HIV^+ nonsmokers. The number of migratory neutrophils were counted after 1 hr of incubation.

Supplemental Figure 9. Isolation of Neutrophils from Peripheral Blood by Negative Selection. Peripheral blood neutrophils were isolated using EasySep Direct Human Neutrophil Isolation

Kit, (StemCell Technologies, Cambridge, MA). Purify of isolated neutrophil was confirmed by flow cytometry analysis of surface expression of CD66b and CD16 (specific markers for neutrophils).

Supplemental Figure 10. Morphology of BC from $HIV^{-}$and HAART-treated HIV^{+} nonsmokers cultured *ex vivo*. Increased number of "holes" were observed in HIV^{+} BC culture.

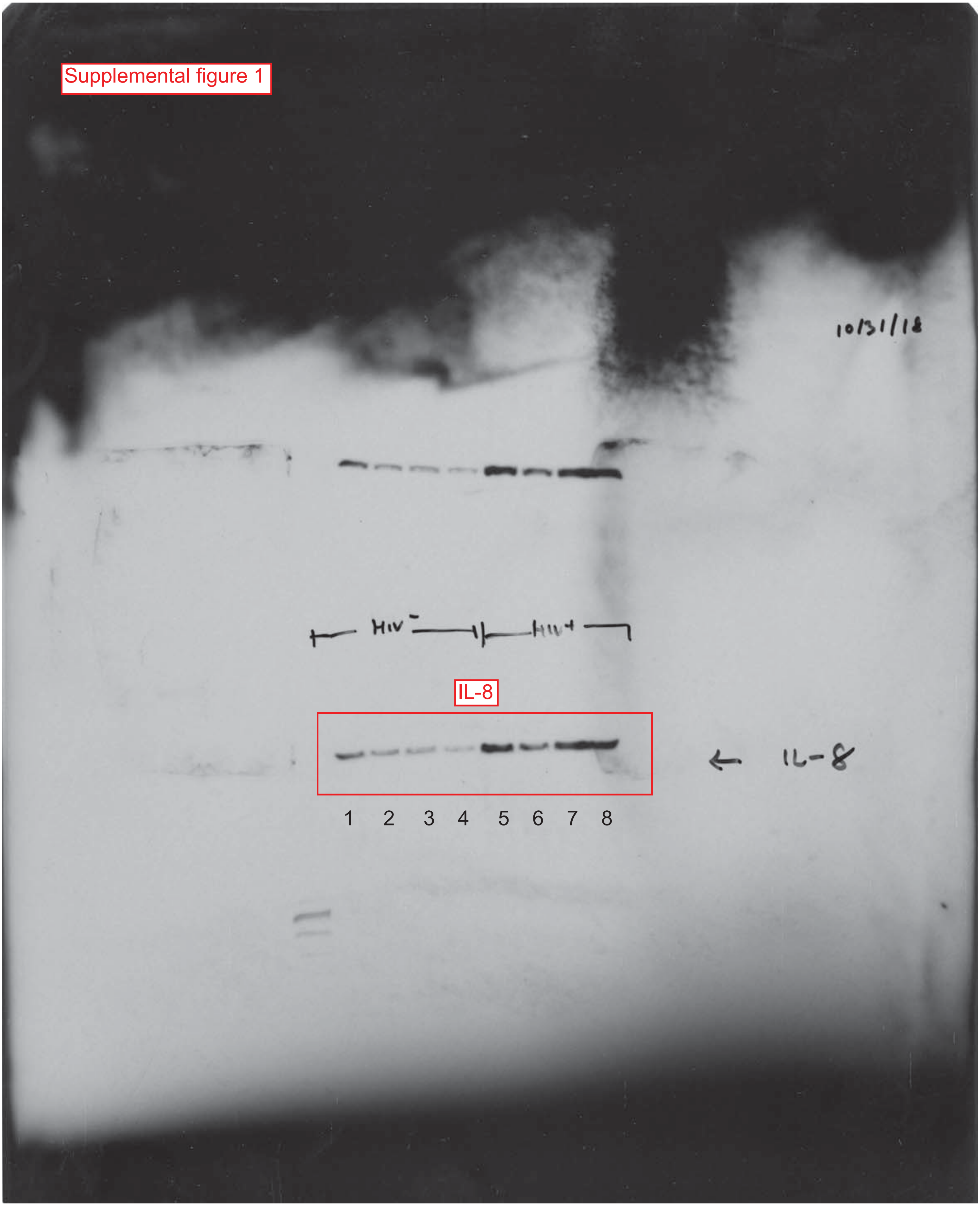
Supplemental figure 1
10/31/18
HIV-
HIV+
IL-8
← IL-8
1 2 3 4 5 6 7 8

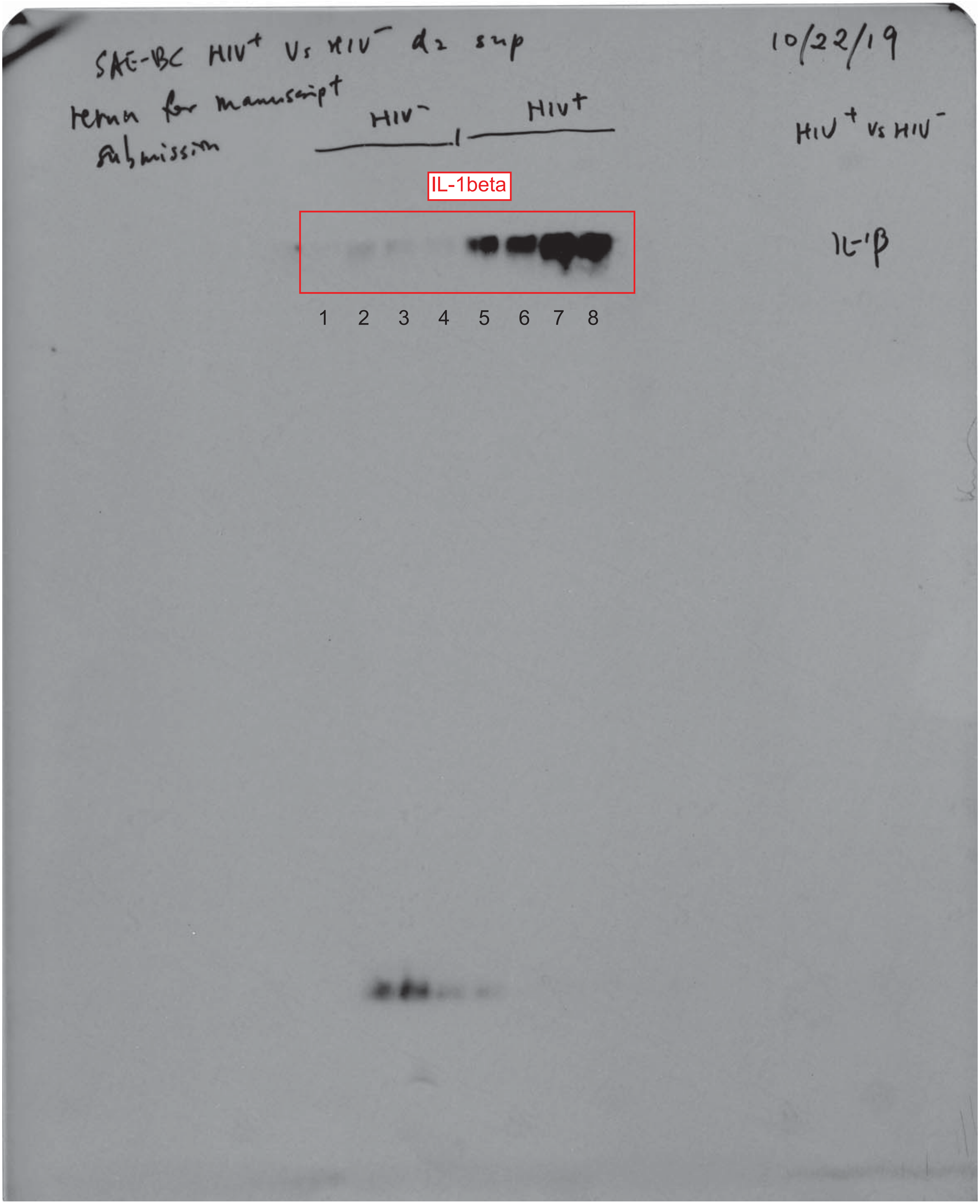
SAE-BC HIV+ vs HIV- d2 sup
10/22/19
rerun for manuscript submission
HIV-
HIV+
HIV+ vs HIV-
IL-1beta
IL-1β
1 2 3 4 5 6 7 8

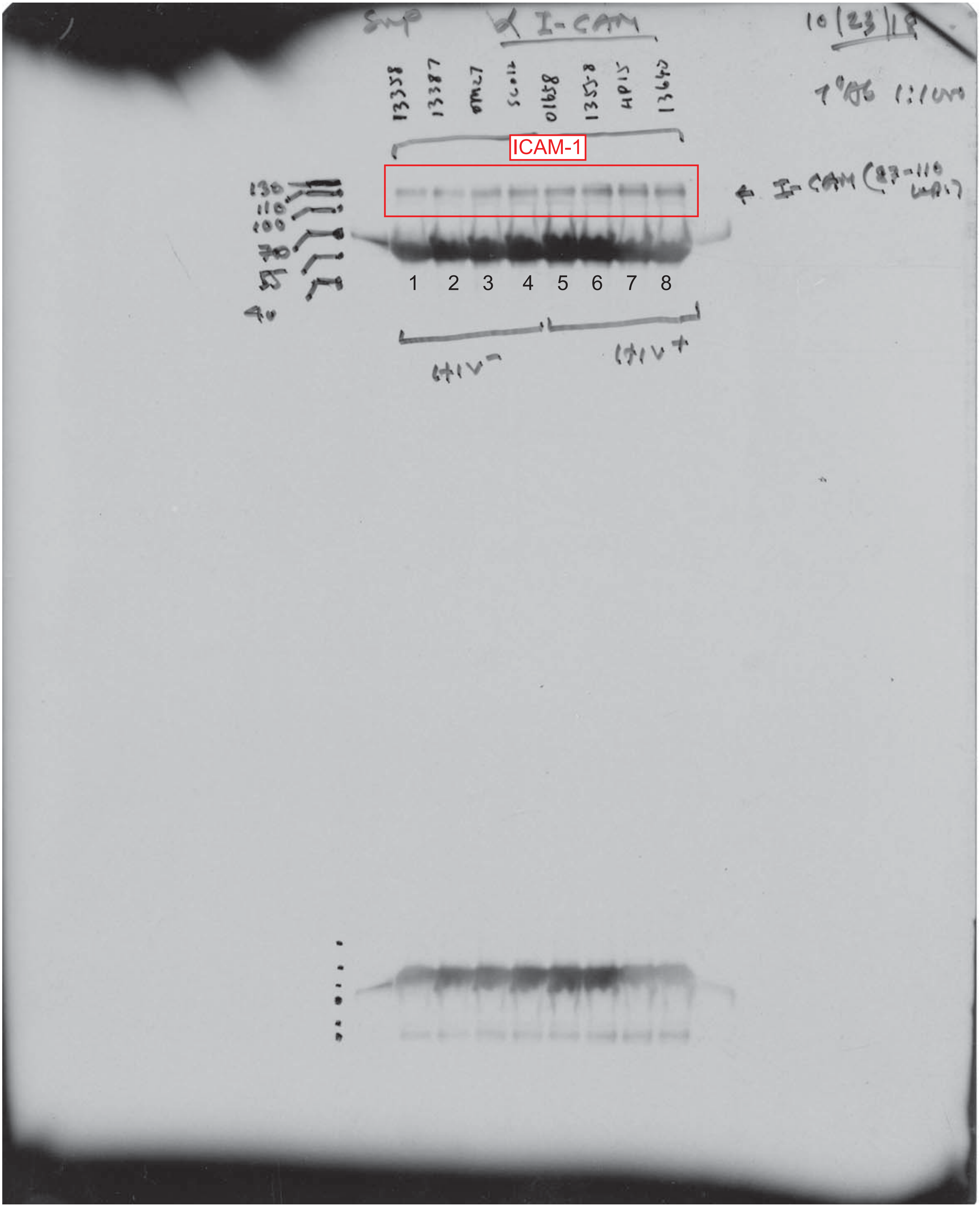

ICAM-1
1 2 3 4 5 6 7 8

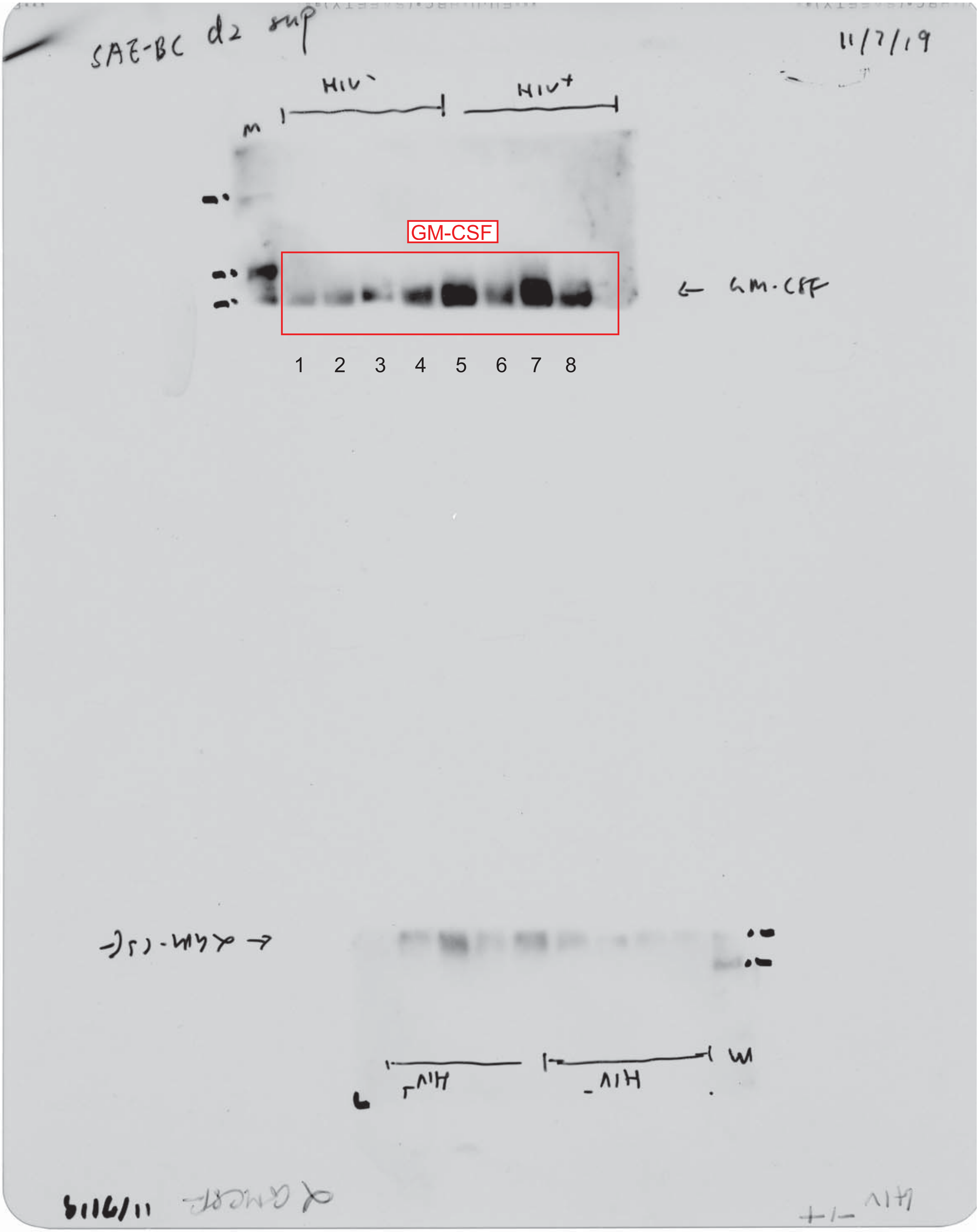
SAE-BC d2 sup
11/7/19
M
HIV-
HIV+
GM-CSF
← GM-CSF
1 2 3 4 5 6 7 8

Supplemental figure 2

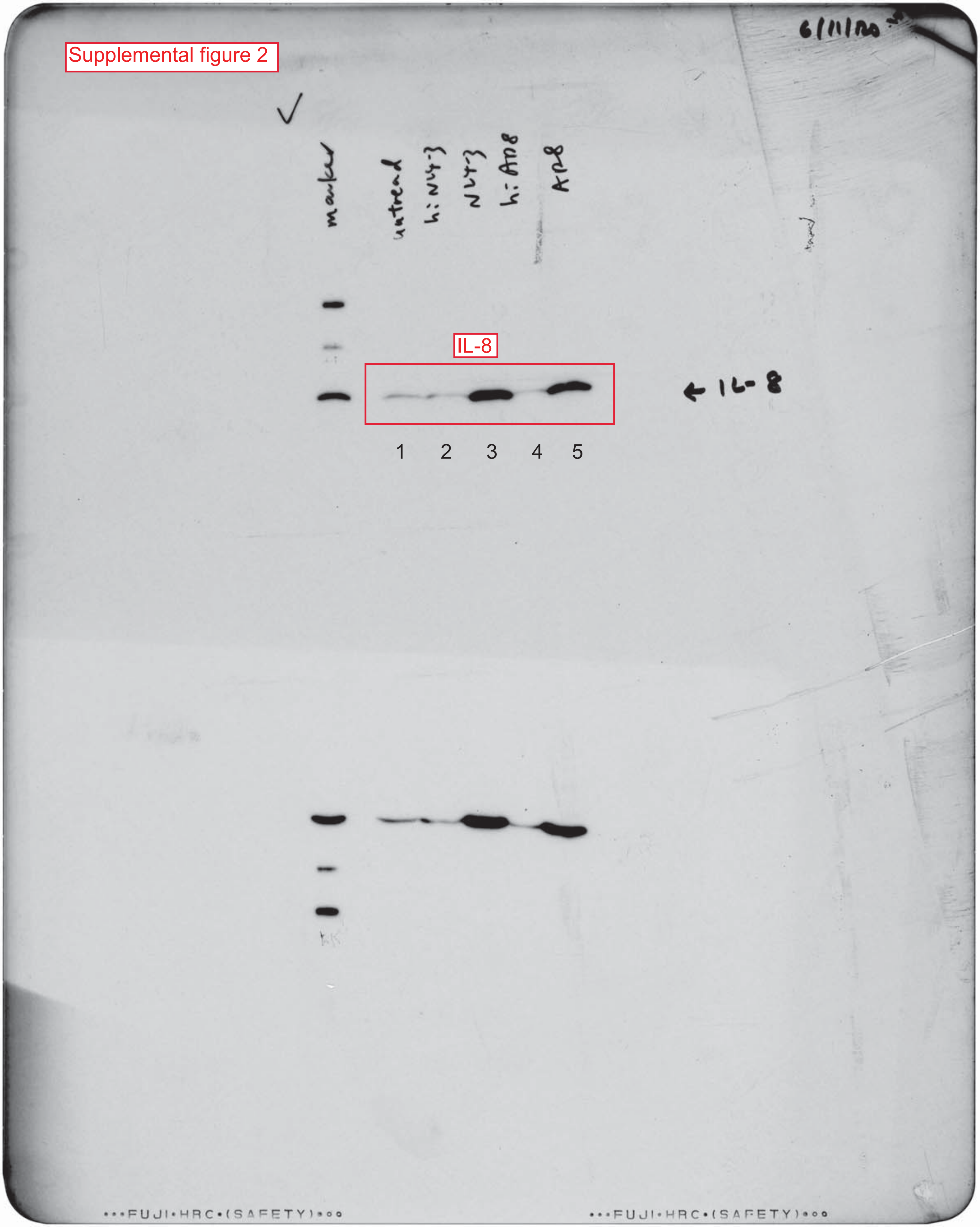

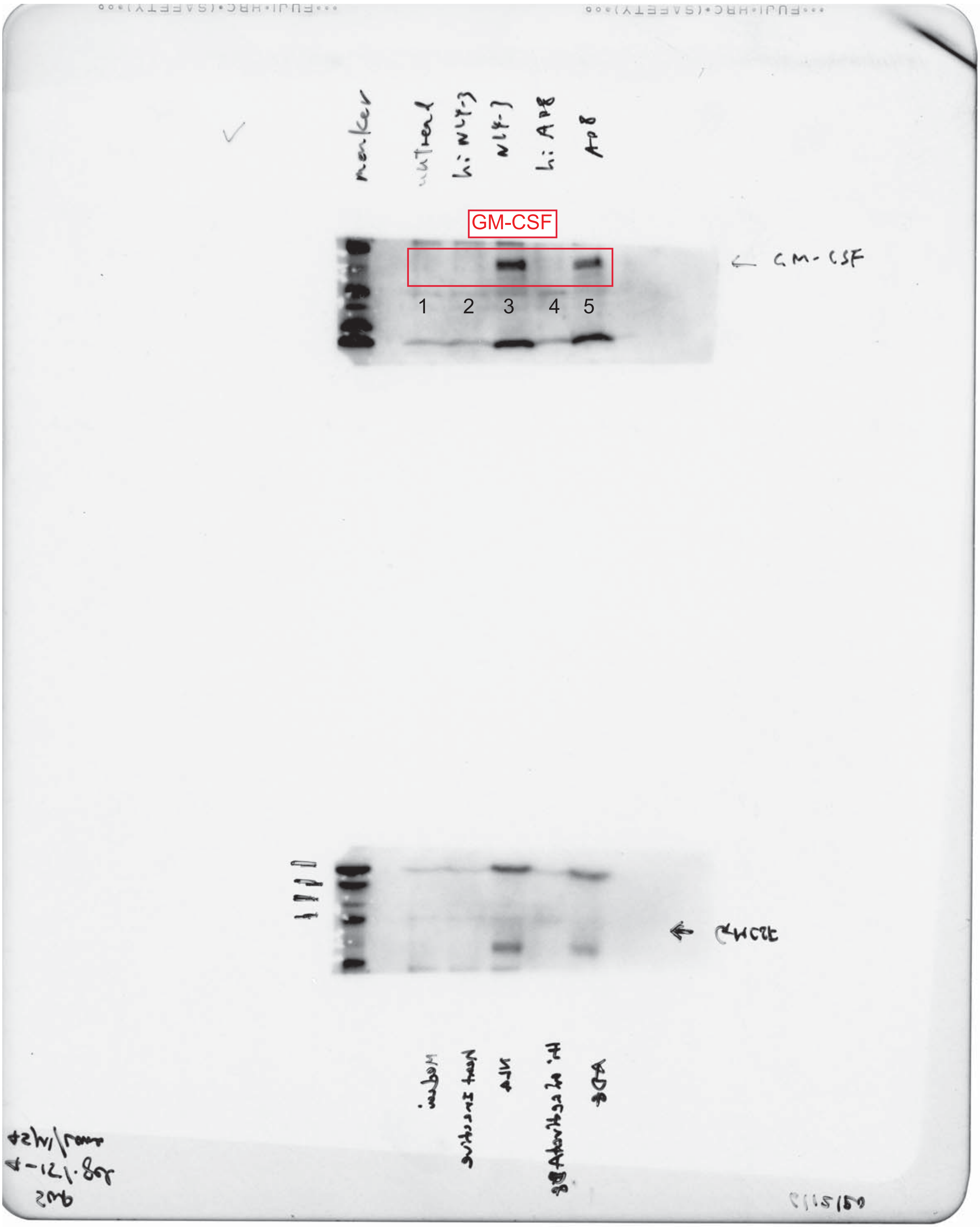

marker
GM-CSF
1
2
3
4
5
← GM-CSF

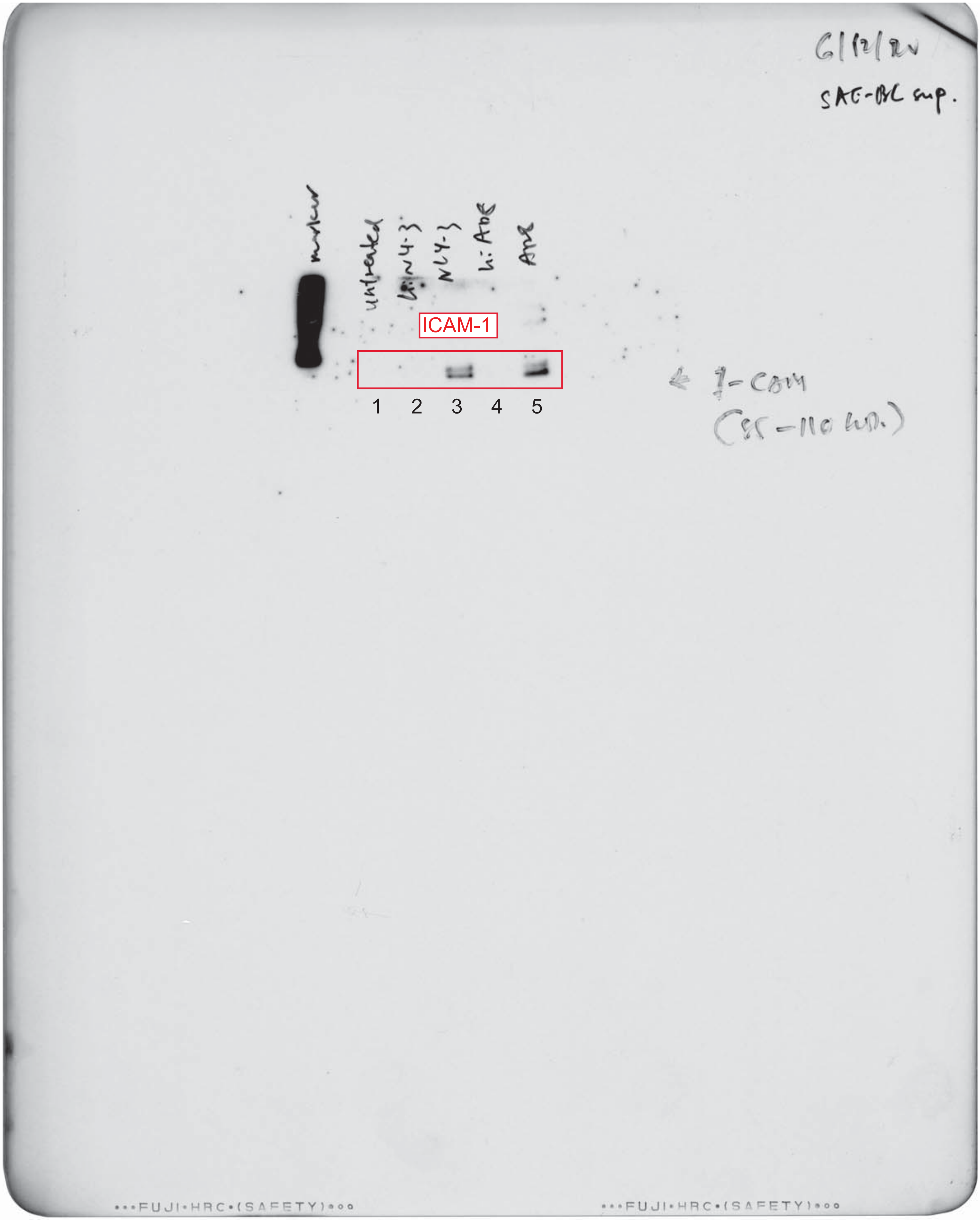
marker
untreated
ICAM-1
1
2
3
4
5
•••FUJI•HRC•(SAFETY)•oo
•••FUJI•HRC•(SAFETY)•oo

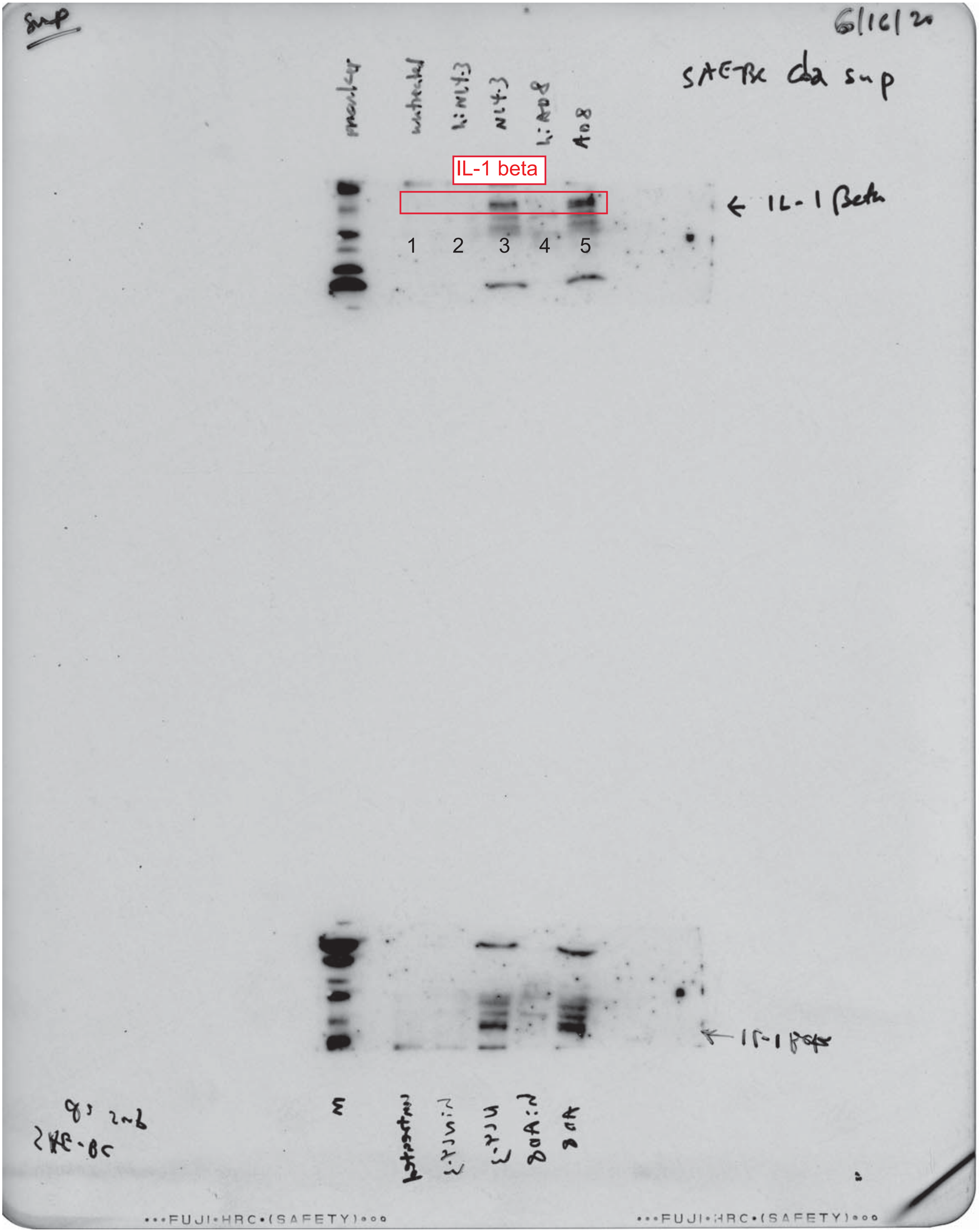
IL-1 beta
1
2
3
4
5
← IL-1 Beta
6/16/20
SAE-BC cda sup
marker
untreated
hi:NL43
NL4-3
hi:Ad8
Ad8
•••FUJI•HRC•(SAFETY)•••
•••FUJI•HRC•(SAFETY)•••

Supplemental Figure 3

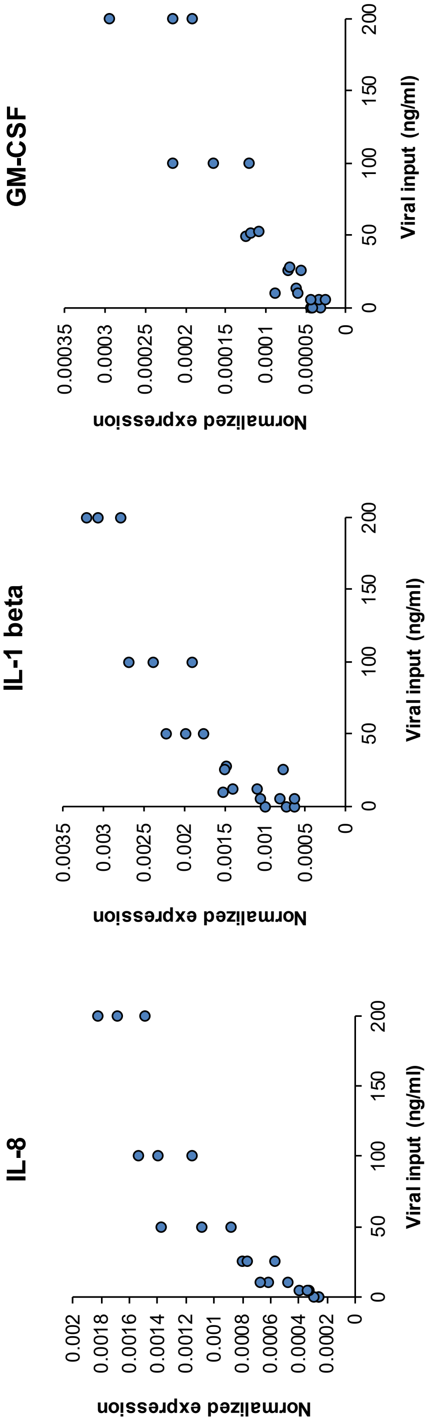

Supplemental Figure 4

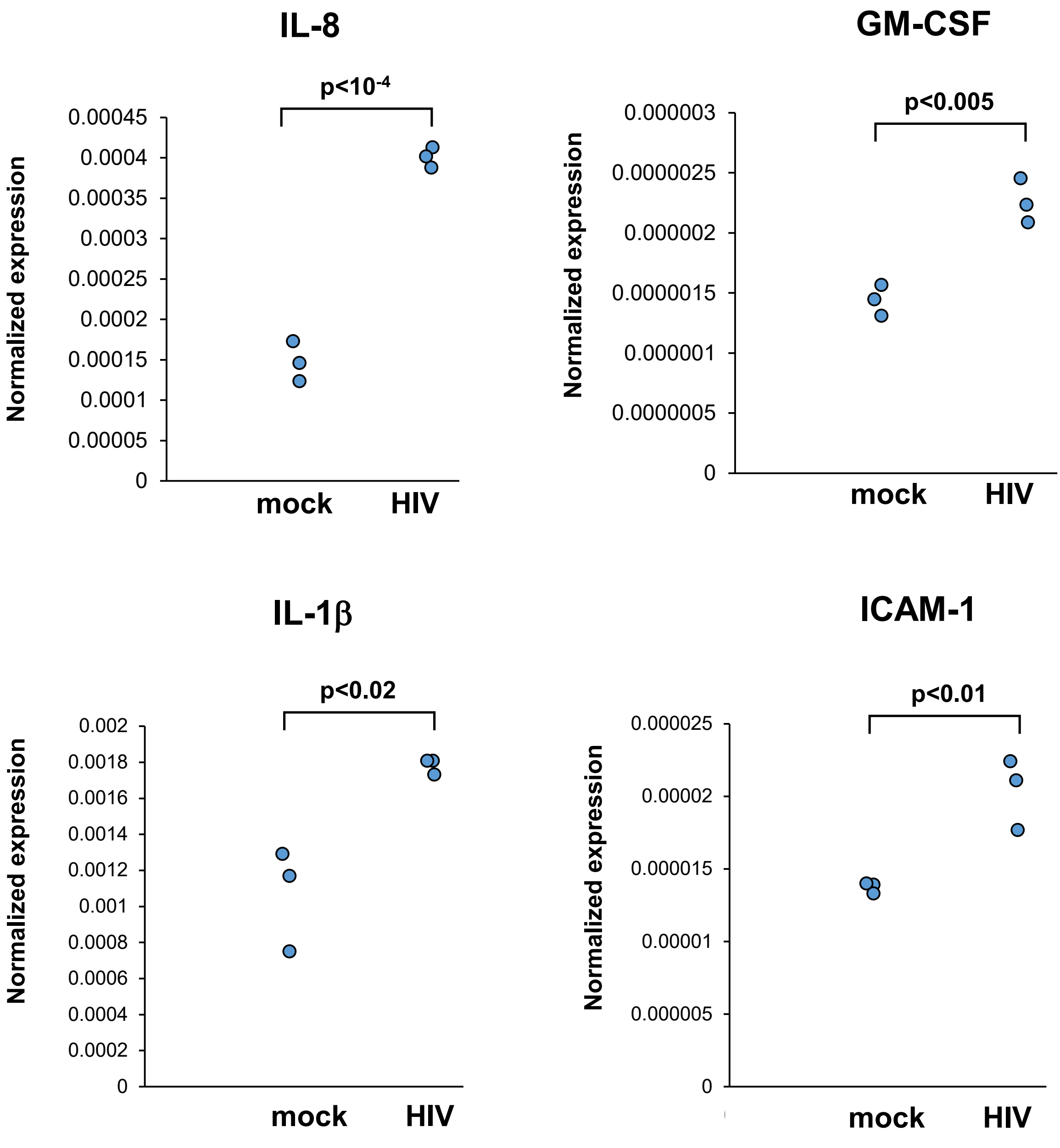

Supplemental Figure 5

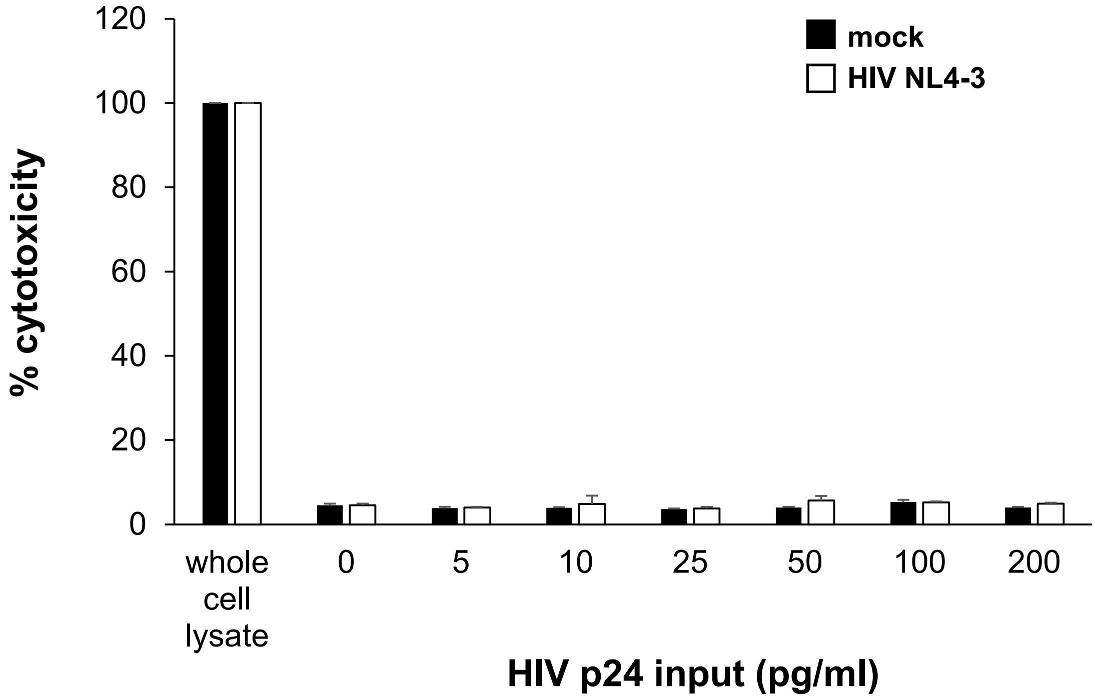

Supplemental Figure 6

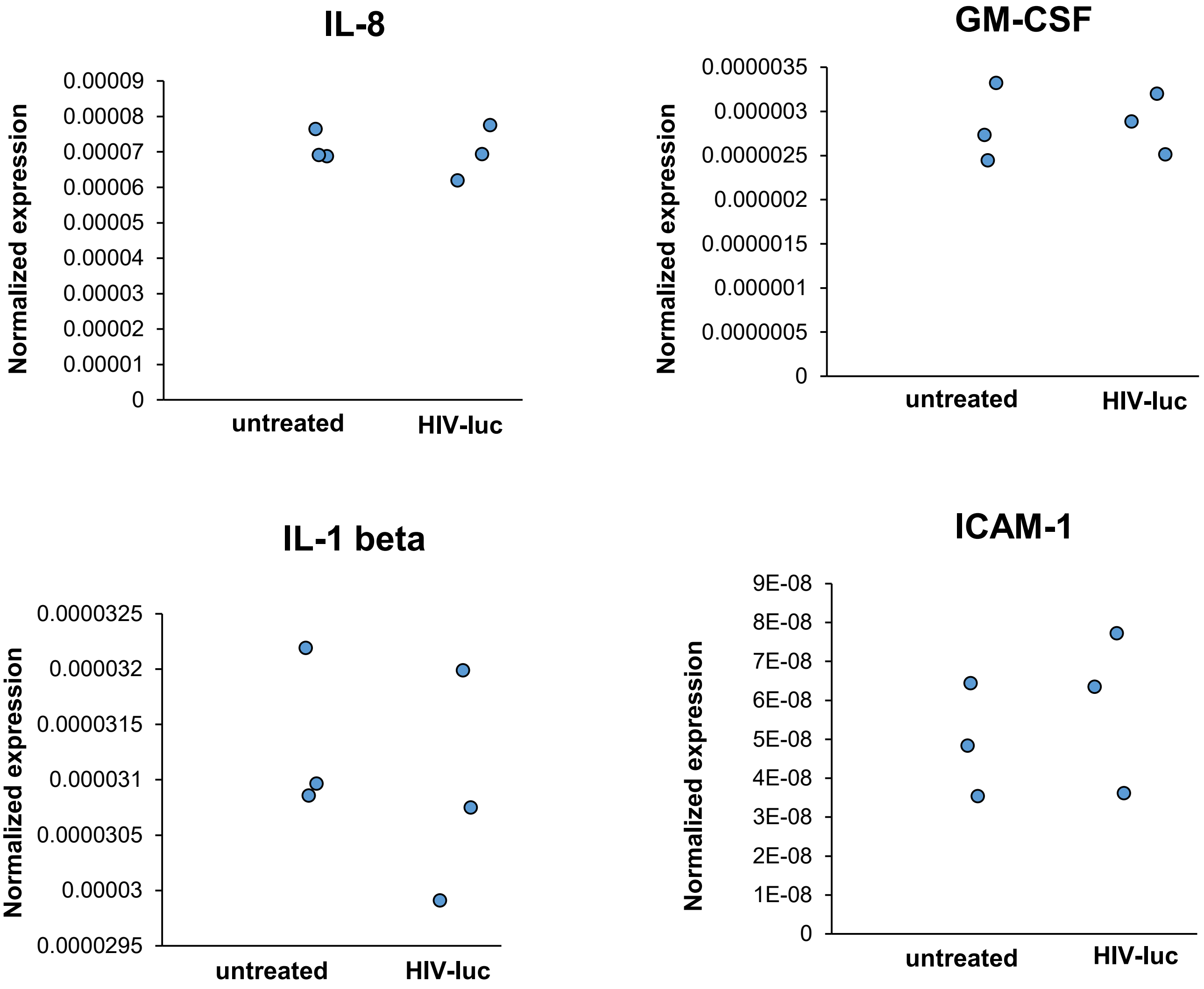

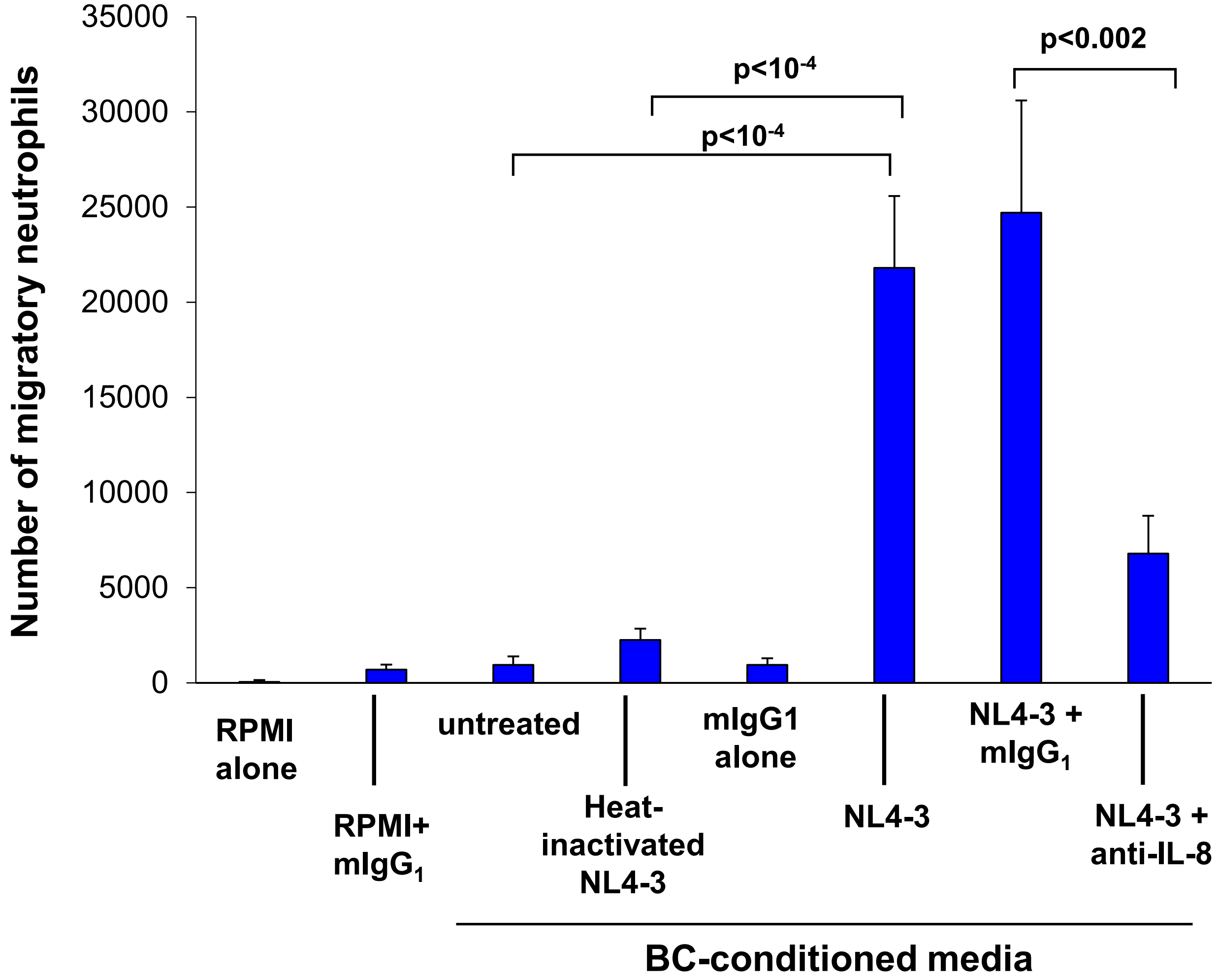
35000
30000
25000
20000
15000
10000
5000
0
Number of migratory neutrophils
p<10-4
p<10-4
p<0.002
RPMI alone
RPMI+ mIgG1
untreated
Heat-inactivated NL4-3
mIgG1 alone
NL4-3
NL4-3 + mIgG1
NL4-3 + anti-IL-8
BC-conditioned media

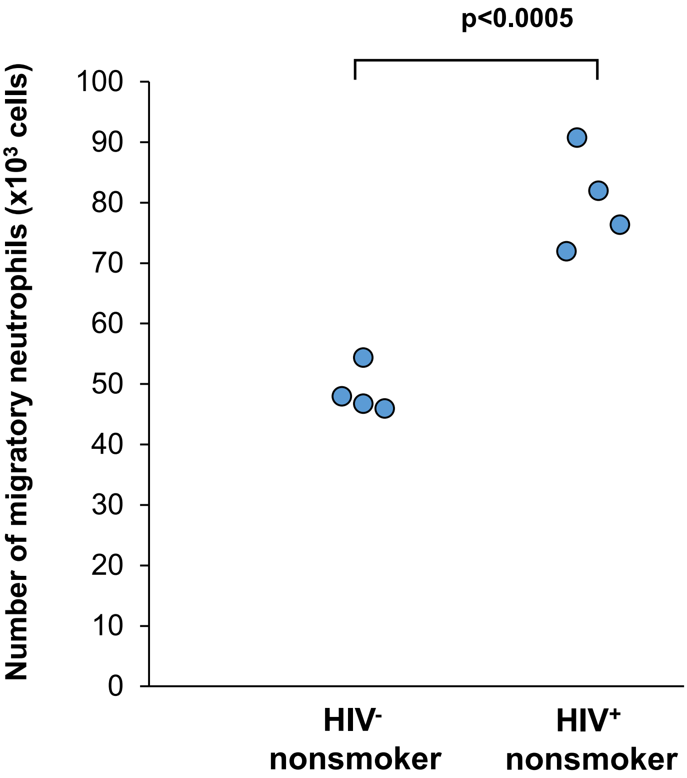
p<0.0005
Number of migratory neutrophils (x10³ cells)
100
90
80
70
60
50
40
30
20
10
0
HIV⁻ nonsmoker
HIV⁺ nonsmoker

Supplemental Figure 9

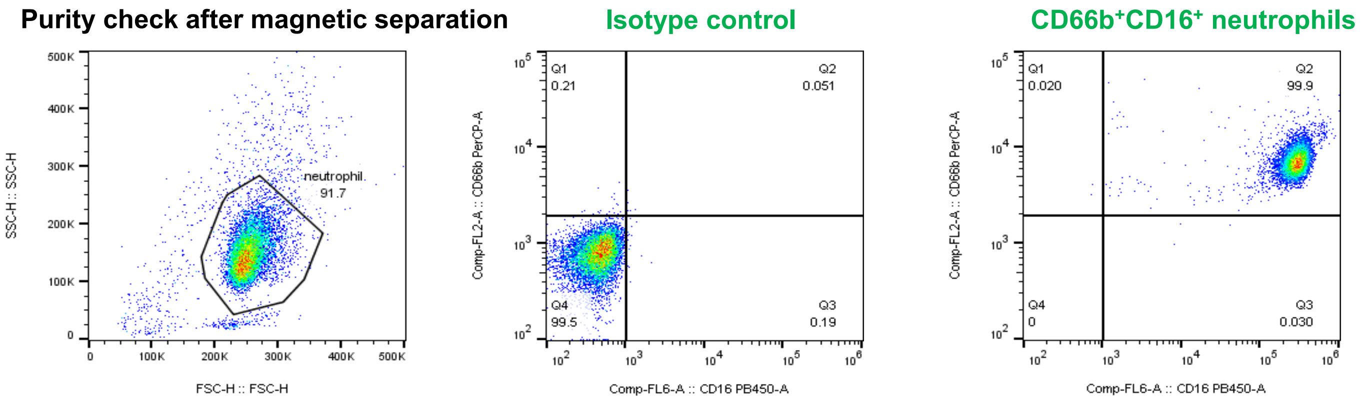

Supplemental Figure 10

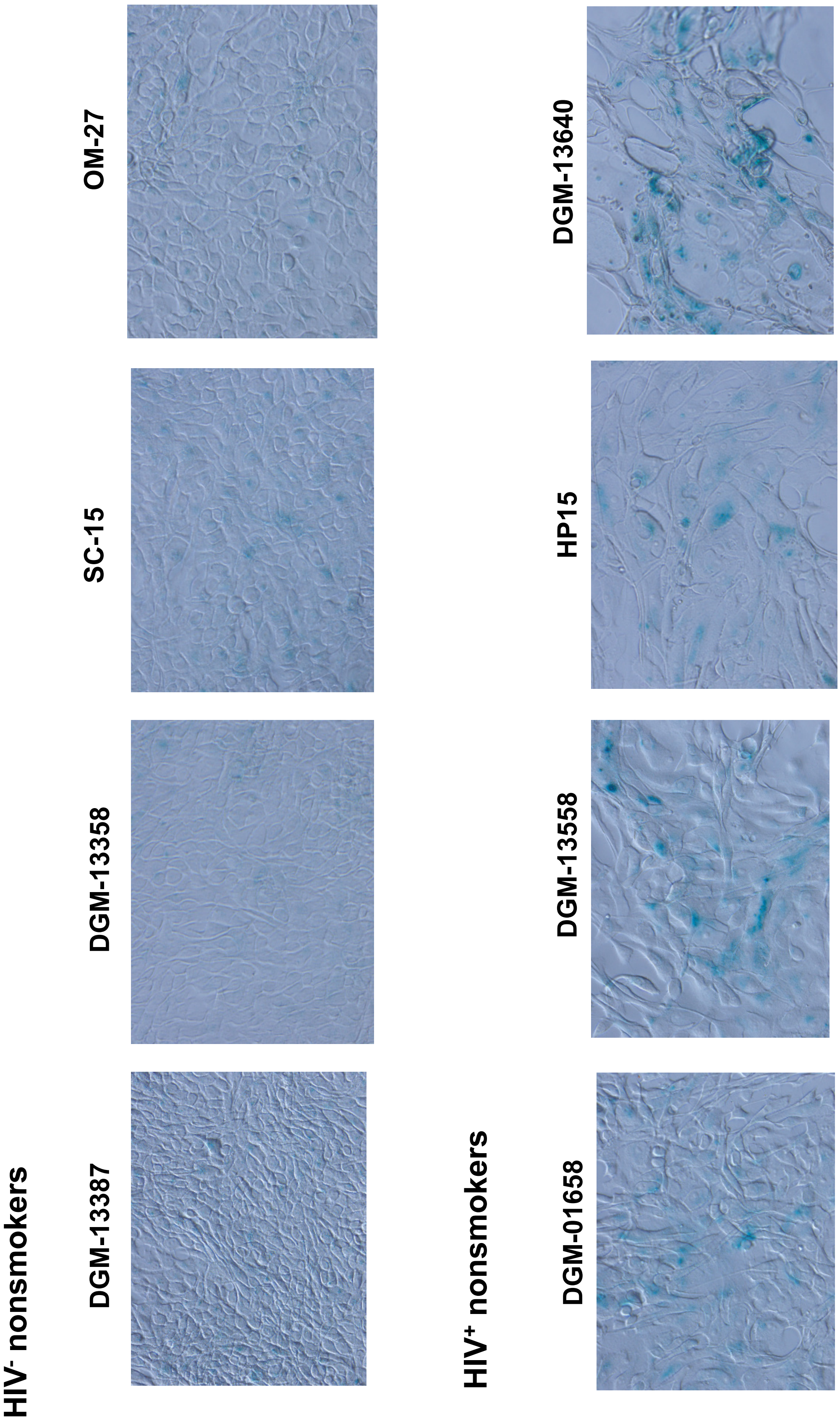

Supplement: Supplementary file 1 — Supplementary Information. [file 41598_2021_82143_MOESM1_ESM.pdf]
